# Transcription Pause and Escape in Neurodevelopmental Disorders

**DOI:** 10.3389/fnins.2022.846272

**Published:** 2022-05-09

**Authors:** Kristel N. Eigenhuis, Hedda B. Somsen, Debbie L. C. van den Berg

**Affiliations:** Department of Cell Biology, Erasmus University Medical Center, Rotterdam, Netherlands

**Keywords:** transcriptional pausing, RNApol2, neurodevelopmental disorders, Cornelia de Lange Syndrome, intellectual disability

## Abstract

Transcription pause-release is an important, highly regulated step in the control of gene expression. Modulated by various factors, it enables signal integration and fine-tuning of transcriptional responses. Mutations in regulators of pause-release have been identified in a range of neurodevelopmental disorders that have several common features affecting multiple organ systems. This review summarizes current knowledge on this novel subclass of disorders, including an overview of clinical features, mechanistic details, and insight into the relevant neurodevelopmental processes.

## Introduction

Gene transcription is a highly regulated process that ultimately determines cellular identity and response to external stimuli. Transcription often occurs in bursts (Wan et al., 2021) and is primarily regulated at the level of burst initiation and promoter proximal RNApol2 (RNA polymerase 2) pausing (Bartman et al., 2019). Pausing takes place following RNApol2 gene entry and recruitment of the general transcription factor TFIIH to the preinitiation complex, which results in melting of the DNA template and rapid progression of RNApol2 to the pause site, 20–120 nucleotides downstream of the TSS (transcription start site). Release from the paused state requires the action of the P-TEFb (Positive Transcription Elongation Factor b) complex, whose kinase module phosphorylates RNApol2 and associated pausing factors to enable entry into productive elongation. Transcription of virtually all genes was shown to be dependent on P-TEFb activity (Jonkers et al., 2014) and significant accumulation of paused RNApol2 was observed at a fraction of these (Day et al., 2016).

Establishment and in particular release from pausing is a highly regulated process involving multiple factors that often also act in other phases of the transcription cycle. Central to pause-release is P-TEFb recruitment to paused RNApol2 as part of a complex with BRD4 (bromodomain containing protein 4) or the SEC (super elongation complex), assisted by Mediator and the PAF1 (polymerase-associated factor 1) complex (Lu et al., 2016). Moreover, whilst BRD4 is not absolutely required for CDK9 recruitment, it is necessary for the assembly of a productive elongation complex (Winter et al., 2017).

**FIGURE 1:**
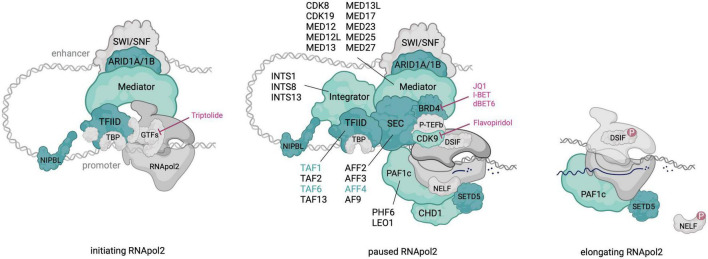
An overview of RNApol2 in initiating, paused and elongating state with transcriptional pausing regulators that contribute to NDDs colored in green. For multi-subunit complexes, subunits with pathogenic NDD variants are outlined. Dark green shading and font highlights factors in which pathogenic variants result in a CdLS-like phenotype. Discussed therapeutics and their targets in the paused RNApol2 complex are indicated. Created with BioRender.com.

Interestingly, pathogenic variants in multiple transcriptional pausing regulators have been identified in neurodevelopmental disorders (NDDs), including CdLS (Cornelia de Lange Syndrome) and CdLS-like disorders (Figure 1). Some of these regulators play a direct role in P-TEFb recruitment (e.g., SEC, BRD4) while others are linked to transcriptional pausing through physical interaction (i.e., NIPBL) or by experimental evidence from cellular systems (e.g., Mediator and PAF1c). Gain-of-function mutations in *AFF4*, encoding a subunit of the SEC, result in the CdLS-related CHOPS (OMIM# 616368) (Cognitive development and coarse facies, Heart defects, Obesity, Pulmonary involvement, and Short stature and skeletal dysplasia) syndrome (Izumi et al., 2015). Heterozygous loss-of-function (LoF) mutations in *BRD4* similarly result in a CdLS-like syndrome (Olley et al., 2018) and NIPBL, whose disruption is the most frequent genetic cause of CdLS, was linked to transcriptional pausing *via* its interaction with the Integrator complex (van den Berg et al., 2017). Mutations in subunits of the Mediator and PAF1 complex have been identified in several intellectual disability (ID) syndromes and haploinsufficiency of SETD5, one of the most frequent genetic causes of idiopathic ID, was recently linked to pausing defects (Deliu et al., 2018). These observations causally link transcriptional pausing defects to NDDs well beyond CdLS.

In this review we will present current insights into the mechanism and gene regulatory implications of RNApol2 pausing and discuss in detail the involvement of specific pausing regulators in the aetiology of several NDDs.

## Mechanistic Overview of Promoter Proximal Pausing

### Establishment and Maintenance of RNApol2 Pausing

Transcription initiation involves establishment of the preinitiation complex (PIC), comprised of general transcription factors (GTFs) and RNApol2. TFIIH, the last GTF to be recruited to the PIC, is required both for initiation and establishment of promoter proximal pausing (reviewed in Chen et al., 2018). Its helicase activity mediates melting of the DNA template to enable open complex formation, while its CDK7 kinase module phosphorylates Ser7 and, notably, Ser5 residues in the RNApol2 CTD (C-terminal domain), resulting in escape from the PIC and progression to the pause site.

RNApol2 pausing is stabilized and shielded from premature termination by the association of DSIF (DRB-sensitivity inducing factor) and NELF (negative elongation factor). Structural studies on the paused elongation complex have shown that NELF stabilizes the paused state by limiting RNApol2 intramolecular mobility and nucleotide triphosphate (NTP) active site entry and by interfering with binding to elongation factors, including TFIIS (Vos et al., 2018b). Diversified roles in the establishment, maintenance, and release of paused RNApol2 have been described for the PAF1 and Integrator complexes, which will be discussed in more detail in the next section.

### Release From Pausing

Release from pausing requires the kinase activity of the P-TEFb complex, consisting of CDK9 and (in most cases) Cyclin T1. CDK9 activity is regulated in a multistep process. First, activation of CDK9 requires phosphorylation of a conserved threonine residue (Thr286) in its T-loop region, primarily catalyzed by TFIIH-subunit CDK7 (Larochelle et al., 2012). Most of the active, nuclear P-TEFb complexes are subsequently sequestered in an inhibitory complex comprised of the 7SK small nuclear (sn) RNA, capping enzyme MePCE, LARP7 and HEXIM1/2 proteins (Barboric et al., 2005; Egloff et al., 2006). Release from the 7SK snRNP (small nuclear ribonucleoprotein) complex is regulated by various enzymatic activities catalyzing post-translational modifications (e.g., HEXIM ubiquitination and Cyclin T acetylation) or modifying the 7SK snRNA structure [reviewed in Bacon and D’Orso (2019)]. Active P-TEFb can then be brought to paused RNApol2 by sequence specific TFs (transcription factors) or as part of a complex with BRD4 or the SEC.

*In vitro*, RNApol2 escape from the paused state requires P-TEFb, PAF1c (PAF1 complex) and the elongation factor SPT6 (Vos et al., 2018a). Active CDK9 phosphorylates NELF, DSIF, and Ser2 residues in the RNApol2 CTD. Phosphorylated NELF dissociates from the paused elongation complex, while phosphorylated DSIF turns into a positive elongation factor that remains associated with elongating RNApol2. Recent studies based on acute depletion of DSIF-subunit SPT5 indeed confirm that this factor plays an essential role in both maintenance of pausing and elongation processivity (Aoi et al., 2021; Hu et al., 2021).

### Role of Pausing in Gene Regulation

RNApol2 pausing was first described for the *Drosophila Melanogaster hsp70* heat shock gene (Gilmour and Lis, 1986; Rougvie and Lis, 1988), where it was thought to enable rapid transcriptional responses to changes in the environment. Genome-wide studies have since shown that it is a widespread phenomenon at all transcribed genes, both in *Drosophila* and mammalian cells (Zeitlinger et al., 2007; Jonkers et al., 2014).

Several roles for RNApol2 pausing in gene regulation have been proposed, as reviewed in Adelman and Lis (2012). Pausing could provide a time window for the association of capping enzymes and elongation factors, ensuring subsequent optimally productive elongation. It was also observed that genes with a high pausing index intrinsically favor nucleosome occupancy over the transcription start site (TSS), suggesting that pausing may contribute to maintenance of a nucleosome free region that enables rapid transcription re-initiation (Gilchrist et al., 2010). Furthermore, by providing an additional level at which gene expression can be controlled, RNApol2 pausing allows for fine-tuning of transcriptional responses through integration of multiple signaling events (Adelman and Lis, 2012). Indeed, computational modeling and specific experimental perturbations showed that biological stimuli impinge on burst initiation and pause release to affect transcriptional output (Bartman et al., 2019). Two independent studies have further linked those two processes by showing that pause duration directly influences transcription initiation rate (Gressel et al., 2017; Shao and Zeitlinger, 2017). Finally, in developing *Drosophila* embryos, strong promoter-proximal pausing contributes to rapid acquisition of transcriptional synchrony required for coordinated responses in tissue development (Lagha et al., 2013).

Estimates on the average length of pause duration vary depending on the chosen model system and methodology. Several studies using inhibitors of transcription initiation or pause-release (i.e., triptolide or flavopiridol) followed by RNApol2 tracking over time determined median pause durations of between 5 and 20 min in *Drosophila* and mouse cells (Henriques et al., 2013; Jonkers et al., 2014; Shao and Zeitlinger, 2017). By introducing a CDK9 analog sensitive mutation, Gressel et al. (2017) could very rapidly and specifically inhibit CDK9 and track its effect on RNApol2 dynamics. Median pause durations measured within the range of 1–2 min. Even shorter pause durations of less than 1 min were detected by fluorescent recovery after photobleaching (FRAP) on GFP-tagged RNApol2 acting in endogenous gene transcription (Steurer et al., 2018) or on an engineered gene array (Darzacq et al., 2007). Interestingly, both these studies also found that a large fraction (∼90%) of paused RNApol2 fails to enter productive elongation and prematurely terminates. These data are important to keep in mind when interpreting downstream effects of pausing deregulation on the steady-state transcriptome.

## TFIID

The TFIID complex consists of TATA box-binding protein TBP and TBP-associated factors (TAFs). It is mostly known for its function as a general transcription factor (GTF) during transcription initiation, where it recognizes several core promoter motifs (e.g., the TATA-box) and, upon promoter binding, recruits RNApol2. Together with other GTFs and RNApol2, the preinitiation complex (PIC) is formed [as reviewed by Roeder (1996)].

However, *in vitro* assays indicate that the presence of TBP is sufficient for PIC formation (Fant et al., 2020), and structural analysis of TFIID shows that it also resides downstream of promoter elements at the pausing site (Cianfrocco et al., 2013; Nogales et al., 2017). Moreover, DNA elements that bind TFIID are enriched at pausing sites in *Drosophila* (Hendrix et al., 2008; Lee et al., 2008; Shao et al., 2019). In line with these results, multiple TAFs have been found to interact with various components of the SEC, including AF9, EAF1 and CDK9 (Biswas et al., 2011; Yadav et al., 2019). *TAF6* knockdown leads to reduced TFIID stability and a loss of interaction with AF9, cyclinT1 and CDK9, resulting in reduced recruitment of TFIID, SEC and RNApol2 to target genes (Yadav et al., 2019). Furthermore, *TAF1* and *TAF2* knockdown causes a widespread increase in transcription at protein coding genes, and a decrease of promoter-proximal pausing (Fant et al., 2020), thus leading to the hypothesis that TFIID may function directly in the regulation of transcriptional pausing.

Being an important transcriptional regulator, TFIID defects are associated with numerous diseases, including cancer (Oh et al., 2017; Xu et al., 2018) and neurodegenerative disease (Makino et al., 2007; Herzfeld et al., 2013; Aneichyk et al., 2018). Several of the TFIID proteins are also associated with neurodevelopmental disorders. The X-linked gene *TAF1* encodes for the largest subunit of TFIID and its mutation is also most frequently described to cause neurodevelopmental delay (Stenson et al., 2014; Niranjan et al., 2015; Hu et al., 2016; Gudmundsson et al., 2019; Kahrizi et al., 2019; Okamoto et al., 2020). Two large studies have described a total of 41 individuals that present with global developmental delay, ID, microcephaly, short stature, characteristic facial dysmorphologies and generalized hypotonia (O’Rawe et al., 2015; Cheng et al., 2019). Two of these individuals were initially diagnosed with CdLS (see below) due to their craniofacial features, growth failure, ID and specific limb malformations. Although there is a large overlap between patient phenotypes, it is hard to distinguish a specific facial gestalt, which could be due to the widespread distribution of *TAF1* missense variants, covering all TAF1 domains (Cheng et al., 2019).

Notably, all disease-causing mutations discovered thus far were either hemizygous or homozygous missense variants, or gene duplications, indicating that TAF1 loss-of-function is potentially lethal. Indeed, *taf1* knockout in zebrafish caused embryonic lethality with deregulated genes enriched for those involved in neurodevelopmental processes (Gudmundsson et al., 2019). In mice and human several ubiquitously expressed TAF1 isoforms have been described, while neuronal tissue expresses an isoform that includes a 6-nucleotide long microexon (Makino et al., 2007). Microexon inclusion is temporally regulated and the resulting neuronal isoform N-TAF1 is predominantly expressed in postmitotic neurons (Capponi et al., 2020). It was postulated by the authors that such cell-type specific splicing events could contribute to tissue-specific disease phenotypes of ubiquitously expressed genes. Whether and how the reported missense variants affect transcriptional pause-release remains to be investigated.

Similar to TAF1, a CdLS phenotype was also discovered in a patient carrying a homozygous missense mutation in *TAF6*, resulting in the first autosomal recessive form of CdLS (Yuan et al., 2015). A second homozygous missense variant causing global developmental delay and syndromic ID was discovered by two independent studies (Alazami et al., 2015; Yuan et al., 2015). Both mutations caused a reduction in interaction of TAF6 with other TFIID subunits (Yuan et al., 2015). A total of ten patients have been identified with four genotypic TAF2 variants, in all cases comprising homozygous missense mutations (Najmabadi et al., 2011; Halevy et al., 2012; Hellman-Aharony et al., 2013; Thevenon et al., 2016; Lesieur-Sebellin et al., 2021). Patients present with global developmental delay, moderate to severe ID, microcephaly and abnormalities in the corpus callosum (reviewed by Lesieur-Sebellin et al., 2021). Furthermore, a single study has identified four patients with *TAF13* mutations from two unrelated families (Tawamie et al., 2017). Similar to *TAF2* and *TAF6* mutation, the disease phenotype is caused by homozygous missense variants. Patients present with developmental delay, mild ID and microcephaly; however, they do not show any dysmorphic facial features. Biochemical and transcriptome analysis on these TAF13 variants indicate a reduced heterodimerization with TAF11, and deregulation of a large set of genes (Tawamie et al., 2017).

Lastly, multiple cases of 6q subtelomeric deletions, characterized by developmental delay, intellectual disability, microcephaly, seizures and dysmorphic features, were linked to the loss of TBP (Eash et al., 2005; Rooms et al., 2006). However, *Tbp*^+/–^ mice do not show significant behavioral abnormalities indicative of cognitive impairment compared to WT mice, while *Tbp^–/–^* mice show very early embryonic lethality (Martianov et al., 2002; Rooms et al., 2006). Nevertheless, discovery of a patient with mild ID, difficulty walking and abnormal movement related to a homozygous deletion resulting in a frameshift in *TBP*, does further indicate a role for TBP in neural development (Monies et al., 2017).

Although not all of TFIID subunits have (yet) been associated with neurodevelopmental disorders, some have been further studied for their impact in neuronal development. *TAF4* is highly expressed in cortical neural stem cells *in vitro*, where it is believed to regulate neuronal differentiation together with intracellular signaling factor RanBPM (Brunkhorst et al., 2005). *Taf4a* knockout mice die at E9.5 and show severe growth retardation, and obvious patterning and morphogenesis defects (Langer et al., 2016). Moreover, *Taf4a^–/–^* ESCs are unable to differentiate into glutamatergic neurons *in vitro* due to impaired PIC formation at differentiation genes (Langer et al., 2016). *Taf9b* is upregulated during neuronal differentiation of mouse ES cells and *Taf9b* knockout causes downregulation of neuronal genes such as *Tubb3*, both *in vitro* and *in vivo* (Herrera et al., 2014).

In summary, the high number of TFIID components found associated with neuronal defects upon mutation or loss indicates that misregulation of TFIID broadly impacts neuronal differentiation. Moreover, the CdLS diagnosis for mutations in TAFs implicated in the regulation of pausing warrants further investigation into this link, as will be discussed below.

## CDK9

CDK9 is widely expressed in all human tissues (De Luca et al., 1997) and plays a role in several diseases, including HIV infection and multiple cancers (Egloff, 2021). In total six patients have been described that carry variants in *CDK9* resulting in CHARGE (coloboma, heart defects, atresia choanae, growth retardation, genital abnormalities and ear abnormalities)-like syndrome (OMIM#214800) (Shaheen et al., 2016; Maddirevula et al., 2019; Nishina et al., 2021). Five of these patients carry homozygous non-synonymous variant p.Arg225Cys and in one patient compound heterozygous missense variants (i.e., p.Ala288Thr and p.Arg303Cys) were detected.

CHARGE syndrome was initially identified in patients with mutations in the chromodomain helicase DNA-binding protein CHD7 and it frequently features intellectual disability and global developmental delay (Vissers et al., 2004). Similarly, the reported CDK9 variants were associated with global developmental delay (5 cases), intellectual disability (2 cases), microcephaly (2 cases), cerebral (3 cases) and cerebellar (3 cases) atrophy, epileptic seizures (3 cases) and myelination defects (1 case) (see also Table 1 for an overview of clinical symptoms). The three affected amino acids are highly conserved amongst vertebrates and locate in the catalytic kinase domain of CDK9. Patient-specific recombinant CDK9 variants showed reduced kinase activity *in vitro*, suggesting that loss of function of CDK9 causes the phenotype (Nishina et al., 2021). To what extent this decrease in enzymatic activity affects RNApol2 pause release remains to be determined.

**TABLE 1:** Overview of clinical symptoms.

			SEC					TFIID					INTS		PAF1					Mediator									
	NIPBL (CdLS)	BRD4 (CdLS)	AFF4 (CHOPS)	AFF3 (KINSSHIP)	AFF2	AF9	CDK9	TAF1	TAF2	TAF6	TAF13	TBP	INTS1	INTS8	PHF6 (BFLS)	LEO1	CHD1 (PILBOS)	SETD5	ARID1A/1B (CSS)	CDK8	CDK19	MED12	MED12L	MED13L	MED13	MED17	MED23	MED25	MED27
**Growth**																													
Prenatal growth deficiency																													
Short stature																													
Microcephaly																													
Macrocephaly																													
**Facial features**																													
Synophrys																													
Brachycephaly																													
Low anterior hairline																													
Arched/thick eyebrows																													
Long eyelashes																													
Ptosis																													
Low set posteriorly rotated ears																													
Anteverted nostrils																													
Depressed nasal bridge																													
Depressed midface																													
Pointed chin																													
Almond-shaped eyes																													
Translucent skin																													
Periorbital fullness																													
Broad nasal tip																													
Long philtrum																													
Broad philtrum																													
Micrognathia																													
Thin upper vermilion (lip)																													
Downturned corners of the mouth																													
highly arched palate																													
Widely spaced/absent teeth																													
High or cleft palate																													
Short neck																													
Prominent glabella																													
Hypertelorism																													
Frontal bossing																													
Dolichocephaly																													
Swelling of subcutaneous tissue of the face																													
Narrow palpebral fissure																													
Large ears																													
Wide mouth																													
Thick lips																													
**Neurology**																													
Cognitive delay																													
Impaired language development																													
Motor impairment																													
Seizures																													
Epilepsy																													
**Cognition and Behavior**																													
Intellectual disability																													
ASD																													
Self-injurious behavior																													
Stereotypic movement																													
**Trunk and limbs**																													
Oligodactyly and adactyly																													
Clinodactyly																													
Small hands																													
Proximally placed thumbs																													
Irregular and overlapping toes																													
Hypoplastic/absent nail of the fifth finger or toe																													
Small feet																													
Hirsutism																													
Vertebral abnormalities																													
Chest or sternum deformity																													
Obesity (with gynecomastia)																													
**Other major systems**																													
Vision defects																													
Gastrointestinal abnormalities																													
Cardiovascular abnormalities																													
Urinary abnormalities																													
Genital abnormalities																													
Renal dysplasia																													
Hypogonadism																													
Hypometabolism																													
Hypotonia																													

The developmental role of CDK9 has been studied in several model organisms. In zebrafish embryos, CDK9 inhibition with flavopiridol or depletion with morpholinos resulted in increased apoptosis and an underdeveloped forebrain and midbrain (Matrone et al., 2016). In mice, homozygous loss of Cdk9 is lethal whereas heterozygous loss causes abnormal morphology of the heart, skin and epididymis (Munoz-Fuentes et al., 2018). P-TEFb was found be required for retinoic acid (RA)-induced neuronal differentiation of neuroblastoma cells (De Falco et al., 2005; Ghosh et al., 2018). Which neurodevelopmental pathways are affected by the reported CDK9 missense variants should be topic of further investigation.

## BRD4

Several factors involved in the recruitment of P-TEFb to paused RNApol2 have been implicated in neurodevelopmental disorders with a CdLS-like phenotype, including BRD4. BRD4 is part of the bromodomain and extra-terminal domain (BET) family, together with BRD2, BRD3 and the testis specific BRDT (Shi and Vakoc, 2014). The bromodomains of these proteins can bind acetylated lysines at histones and transcription factors, mediating their recruitment to active chromatin (Dey et al., 2003; Wu et al., 2013; Shi and Vakoc, 2014).

The BET inhibitors such as JQ1 and I-BET result in chromatin dissociation of BRD4 and subsequent deregulation of global gene expression (Filippakopoulos et al., 2010; Dawson et al., 2011; Xu and Vakoc, 2017). Rapid BET protein degradation by dBET6 resulted in accumulation of paused RNApol2 and a severe loss of Ser2-phosphorylated, elongating RNApol2 (Winter et al., 2017), an effect linked to BRD4 and not BRD2 or BRD3 as shown by degron-based depletion studies (Arnold et al., 2021; Zheng et al., 2021).

BRD4 contains a unique interaction domain for P-TEFb (Bisgrove et al., 2007) and interacts with other important pausing factors such as the Mediator complex (Jiang et al., 1998; Jang et al., 2005; Wu and Chiang, 2007), the PAF1 complex, and DSIF (Yu et al., 2015; Arnold et al., 2021). A systematic analysis in HeLa cells has shown that BRD4 recruits P-TEFb specifically to DSIF-subunit SPT5 (Lu et al., 2016). However, BET protein degradation or targeted BRD4 depletion did not impact chromatin association of P-TEFb (Winter et al., 2017; Muhar et al., 2018). This observation supports the hypothesis that BRD4 functions as an allosteric activator of P-TEFb, allowing it to work efficiently once in proximity to the paused complex (Schroder et al., 2012; Itzen et al., 2014).

Other roles of BRD4 in the regulation of transcription have also been described. For example, BRD4 can facilitate elongation independently of P-TEFb (Kanno et al., 2014). The ET domain of BRD4 interacts with several factors to drive transcription activation (Rahman et al., 2011) and BRD4 can function as an atypical kinase to phosphorylate Serine 2 in the CTD of RNApol2 *in vitro* (Devaiah et al., 2012). BRD4 specific and pan-BET protein degradation, resulting in widespread transcription continuation downstream of the termination zone, suggested a role for BRD4 in 3′-processing and transcription termination (Arnold et al., 2021). Lastly, BRD4 levels are particularly high at super-enhancers (SEs) where, together with MED1, it is described as a component of liquid–liquid phase separated transcriptional condensates (Boija et al., 2018; Sabari et al., 2018).

Heterozygous, multigenic deletions in chromosome 19, encompassing *BRD4*, have been linked to intellectual disability in multiple probands (Jensen et al., 2009; Bonaglia et al., 2010; van der Aa et al., 2010; Gallant et al., 2011; Jelsig et al., 2012; Olley et al., 2018; Alesi et al., 2019). Moreover, recent studies identified four patients with intragenic mutations in BRD4, resulting in a CdLS-like phenotype characterized by intellectual disability, microcephaly, developmental delay, and many of the CdLS facial features (Olley et al., 2018; Rentas et al., 2020). Mutations included two non-sense variants and two missense variants in the second bromodomain of BRD4, leading to impaired chromatin-association. In mice, heterozygous loss of Brd4 leads to early postnatal mortality, severe prenatal growth failure, reduced body fat, and abnormalities of the craniofacial skeleton (Houzelstein et al., 2002). These features are also commonly found in CdLS, suggesting BRD4 haploinsufficiency and, by extension, deregulated transcriptional pausing as likely cause of the CdLS-like phenotype.

Other functional effects of BRD4 haploinsufficiency besides transcriptional imbalance have also been proposed as alternative causes of the CdLS-like phenotype. Impaired regulation of DNA repair but not transcription was found in BRD4 mutated mESCs and in CdLS patient lymphoblastoid cells (LCLs) (Olley et al., 2018). Similarly, CdLS cells show increased DNA damage sensitivity (Vrouwe et al., 2007). Proper DNA repair is imperative for neural development (Frank et al., 2000; Gao et al., 2000) and mutation of proteins involved in DNA damage repair are often associated with neurodevelopmental defects [reviewed in Lee et al. (2016)], suggesting that defective DNA repair may also contribute to the CdLS-like phenotype of BRD4 heterozygous LoF patients.

Several studies have addressed BRD4 function in the central nervous system. In the adult mouse brain, *Brd4* is predominantly expressed in neurons, where it regulates immediate early gene (IEG) expression (Korb et al., 2015). Rapid induction of IEGs in response to neuronal activity relies on the presence of promoter proximal paused RNApol2 (Saha et al., 2011). IEGs are essential for consolidation of synaptic modification and memory function (Frey et al., 1989, 1996; Nguyen et al., 1994; Messaoudi et al., 2002). Consequently, BET protein inhibition with JQ1 reduced expression of synaptic proteins and resulted in long-term memory deficits (Korb et al., 2015). Together, this suggests a critical role for BRD4 in transcription regulation and neuronal activation during memory formation.

Dysregulation of BRD4 has been causally linked to Rett Syndrome (Xiang et al., 2020) and fragile X Syndrome (FXS) (Korb et al., 2017), two of the most prevalent neurodevelopmental disorders. FXS, caused by loss of the translation repressor FMRP (fragile X mental retardation protein) is characterized by intellectual disability, behavioral deficits, and autism spectrum disorder (ASD). *Brd4* transcripts were identified as direct targets of FMRP, resulting in elevated Brd4 protein levels in *Fmr1* knockout mice. Treatment of these FXS modeling mice with JQ1 reversed aberrant neuronal spine density and gene expression, as well as atypical social and repetitive behavior. Similar beneficial effects of JQ1 were observed in human and mouse models of Rett syndrome (RTT), caused by loss of function of the X-linked gene encoding MeCP2 (methyl-CpG binding protein 2). Increased chromatin binding of BRD4 in *in vitro* differentiated human RTT interneurons, and in MGE (medial ganglionic eminence) and cortex mimicking RTT organoids, caused extensive transcriptional dysregulation that was reverted upon exposure to JQ1. Importantly, JQ1 treatment of RTT modeling *MeCP2^–/Y^* mouse pups improved short term survival and slowed down phenotypic progression.

Collectively, these studies highlight the importance of BRD4 dosage during neurodevelopment. They also underscore the feasibility of postnatal phenotypic reversal of some aspects of NDDs and suggest that at least part of the pathology results from aberrant gene regulation in fully differentiated postmitotic neurons. Rebalancing of transcription pause regulation and elongation can thus be used as a therapeutic strategy to ameliorate symptoms related to NDDs.

## NIPBL

NIPBL (Nipped-B-like) encodes for the protein delangin, the human homolog of fly Nipped-B protein and fungal sister chromatid cohesion protein 2 (SCC2), which together with SCC4 forms a complex that is necessary for cohesin loading onto chromosomes. Recent studies physically and functionally linked NIPBL to the regulation of transcriptional pausing (van den Berg et al., 2017; Olley et al., 2018; Luna-Pelaez et al., 2019). Here, the diverse roles of NIPBL in gene and chromatin architecture regulation will be considered in the context of neural development.

Gene variants in cohesin core components and regulatory proteins are identified as the cause of CdLS (OMIM# 122470, 300590, 300882, 610759, and 614701), a dominant and genetically heterogeneous neurodevelopmental disorder with physical, cognitive and behavioral characteristics (Kline et al., 2018). CdLS prevalence is estimated to be around 1:10,000 – 1:30,000 live births (Kline et al., 2007). Characteristic features include craniofacial anomalies, intellectual disability, psychomotor delay, pre- and postnatal growth retardation, upper limb malformations, hirsutism, and affected gastrointestinal and visceral organ systems (overview of clinical symptoms in Table 1).

Heterozygous LoF or missense variants in NIPBL are identified in approximately 70% of cases whereas variants in SMC1A, SMC3, RAD21, and HDAC8 account for another 5% of (non-)classic cases with overlapping and often milder phenotypes (Liu and Baynam, 2010; Ansari et al., 2014). Heterozygous NIPBL LoF variants are localized throughout the coding sequence and associate with more severe phenotypes, while milder missense variants locate predominantly to important functional domains at the interface with DNA, MAU2, RAD21, SMC1, and SMC3 (Mannini et al., 2013; Shi et al., 2020). Compensatory expression from the intact *NIPBL* allele is frequently observed and a reduction of ∼15% in expression is enough to observe a clinical phenotype (Deardorff et al., 1993). Furthermore, somatic mosaicism for *NIPBL* mutations is reported in 10–23% of ‘classic CdLS’ diagnosed patients (Huisman et al., 2013; Braunholz et al., 2015; Latorre-Pellicer et al., 2021).

Consistent with the function of NIPBL as cohesin loading factor, CdLS patient-derived NIPBL^+/–^ lymphoblastoid cells (LCLs), *Nipbl^+/^*^–^ mouse embryonic fibroblasts (MEFs), and fetal liver cells exhibit reduced global or local cohesin binding and defective 3D genome organization (Liu and Krantz, 2009; Chien et al., 2011; Newkirk et al., 2017). Formation of such chromatin loops by loop extrusion relies on an active holoenzyme consisting of cohesin and NIPBL-MAU2 (Davidson et al., 2019; Kim et al., 2019).

Although reduced Nipbl levels in a CdLS mouse model did not affect bulk cohesin loading, deregulated genes showed reduced cohesin binding (Remeseiro et al., 2013). In the absence of overt chromosome segregation defects (Castronovo et al., 2009), deregulated gene expression likely underlies neuronal dysfunction in CdLS (Castronovo et al., 2009; Kawauchi et al., 2009; Remeseiro et al., 2013). In addition, NIPBL recruits cohesin to sites of double-strand breaks (DSBs) for DNA repair under control of MDC1, RNF168 and HP1y (Oka et al., 2011).

A direct link to gene regulation was established for Nipped-B, the fly homolog of delangin, which was found to regulate Notch signaling and other developmental pathways by facilitating enhancer-promoter communication (Krantz et al., 2004; Tonkin et al., 2004). In mammalian cells, Zuin et al. (2014) subsequently showed that NIPBL binds to promoters of active genes to regulate their expression, independent of cohesin. In addition, there are many transcription factors amongst the NIPBL target genes that are differentially expressed in CdLS. These findings indicate that NIPBL influences transcription in several ways; by loading cohesin complexes that regulate genes *via* chromatin insulation and chromosomal long-range interactions, directly by binding at gene promoters and indirectly through regulation of TF expression.

NIPBL was linked to the regulation of transcriptional pausing *via* its interaction with the Integrator complex in mouse neural progenitor cells (van den Berg et al., 2017). NIPBL genomic binding was enriched at promoters containing paused RNApol2 and important for the regulation of neuronal migration genes (e.g., *Sema3a, Nrp1, Plxnd1*, and *Gabbr2*) and consequently for normal cortical neuron migration *in vivo*. Defects in neuronal migration and subsequent aberrant neuronal positioning disrupt neural circuit formation and have been causally linked to intellectual disability and seizures, both features of CdLS (Liu and Krantz, 2009).

Evidence for a role of NIPBL and cohesin in transcriptional pausing has also been found in *Drosophila*, where promoter-proximal cohesin binding correlated with a significantly higher pausing index that was similarly affected by Nipped-B or Rad21 depletion (Fay et al., 2011; Schaaf et al., 2013). Taken together, these findings implicate transcriptional pausing defects in the aetiology of CdLS, a hypothesis further supported by the causal linkage of variants in bona-fide pausing regulators BRD4, AFF4 and Integrator complex to CdLS-like disorders, as discussed in other sections of this review.

To what extent NIPBL function in gene regulation can be uncoupled from cohesin function remains unclear. Widespread NIPBL binding in the absence of cohesin was detected at active promoters and enhancers in mouse embryonic fibroblasts, neural progenitor cells, and lymphoblastoid cells (Zuin et al., 2014; Busslinger et al., 2017; van den Berg et al., 2017). However, the recently solved cryo-EM structure of the fission yeast and human cohesin-NIPBL-DNA complex suggests that the NIPBL-MAU2 loading complex forms an integral part of DNA-bound cohesin (Higashi et al., 2020; Shi et al., 2020). In addition, *in vitro* reconstitution assays show that NIPBL-MAU2 is required for cohesin-mediated loop extrusion (Davidson et al., 2019; Kim et al., 2019).

In support of a role for the NIPBL-MAU2-cohesin holoenzyme in gene regulation, Weiss et al. (2021) recently reported significant overlap in deregulated genes between patient-derived, NIPBL haploinsufficient cortical neurons and mouse postmitotic neurons acutely depleted of RAD21. Deregulated genes were enriched for neuronal functions related to signaling processes, synaptic transmission, learning and behavior. Disrupted 3D genome organization and transcriptional control in these post-mitotic cortical mouse neurons furthermore emphasized that cohesin is continuously required for neuronal gene expression. Further research is required to answer the question whether NIPBL variants cause deregulated gene expression in CdLS directly, *via* dysregulated pausing, *via* reduced cohesin function, or both.

## Super Elongation Complex

The super elongation complex (SEC) comprises various elongation factors and incorporates active P-TEFb. The AF4/FRM2 family (AFF) forms the scaffold; the canonical SEC contains either AFF1 or AFF4, whereas SEC-like complex 2 and 3 contain AFF2 or AFF3, respectively. These scaffolding proteins interact with 11—19 lysine-rich leukemia (ELL) 1, ELL2 or EEL3, the ELL-associated factor EAF1 or EAF2 and eleven-nineteen leukemia (ENL) or AF9 (He et al., 2010; Lin et al., 2010, 2011; Smith et al., 2011).

Initially, many of the SEC components were identified as translocation partner of the mixed lineage leukemia (MLL) gene (Thirman et al., 1994; Lin et al., 2010). Upon MLL-fusion, SEC is recruited to MLL target genes where it promotes transcription elongation (Mueller et al., 2009; Lin et al., 2010; Yokoyama et al., 2010). This role can be directly linked to its association with P-TEFb. In addition, SEC interacts with important pausing factors such as Mediator (Takahashi et al., 2011; Lens et al., 2017), PAF1c (Kim et al., 2010; He et al., 2011; Wier et al., 2013) and the Integrator complex (Gardini et al., 2014). PAF1c and Mediator recruit SEC to phosphorylate NELF subunits and the RNApol2 CTD, resulting in release of NELF and entry into elongation (Lu et al., 2016). Acute degron-based AFF4 depletion resulted in increased promoter-proximal pausing and decreased RNApol2 in the gene body of heat shock induced genes (Zheng et al., 2021), confirming its role in transcription pause release.

The SEC has been found to regulate expression of many IEGs and developmental control genes involved in neuronal lineage commitment, such as *HOX* genes (Yokoyama et al., 2010; Lin et al., 2011; Luo et al., 2012). Dosing SEC-activity in *Drosophila* neuroblasts is essential to maintain the right balance between self-renewal and differentiation (Liu et al., 2017). Perhaps unsurprisingly, mutations in SEC subunits lead to neurodevelopmental syndromes such as Fragile XE ID, CHOPS and KINSSHIP syndrome (Pramparo et al., 2005; Striano et al., 2005; Izumi et al., 2015; Voisin et al., 2021).

### AFF4

AF4/FRM2 family member 4 (AFF4) is essential for SEC stability and proper transcription in metazoans (He et al., 2010; Lin et al., 2010). Initially, three patients with a CdLS-like phenotype (intellectual disability, short stature and craniofacial dysmorphism) were identified that carried missense mutations in *AFF4*. Absence of certain typical CdLS features, including microcephaly, lead to the delineation of a novel syndrome called CHOPS for Cognitive impairment, Coarse facies, Obesity, Pulmonary involvement, Short stature and skeletal dysplasia (OMIM# 616368) (Izumi et al., 2015).

Currently, 12 individuals have been identified with mutations in *AFF4* leading to CHOPS syndrome (Izumi et al., 2015; Raible et al., 2019; Kim et al., 2021). In all cases, a missense mutation was found in the highly conserved ALF homology domain (Bitoun and Davies, 2005), which interacts with the E3 ubiquitin ligase SIAH1 to regulate AFF4 protein stability (Oliver et al., 2004). Increased AFF4 protein stability and chromatin association, resulting in upregulation of transcriptional targets also found upregulated in CdLS, has been proposed as the causative mechanism (Izumi et al., 2015).

### AFF3/LAF4

Lymphoid nuclear protein related to AF4 (LAF4), also known as AFF3, is also an MLL fusion partner (Ma and Staudt, 1996; von Bergh et al., 2002). Like the other AFF proteins, AFF3 functions as a scaffolding protein for interaction with AF9 or ENL and P-TEFb to form SEC-like 3 (Bitoun et al., 2007; Luo et al., 2012). In this manner, it mediates transcriptional activity through regulation of transcription pausing of a specific subset of genes, including imprinted genes such as *XIST* (Luo et al., 2016; Wang et al., 2017; Zhang et al., 2019). Aff3 overexpression predominantly leads to gene upregulation in the mouse cortex, indicating a positive role in transcription (Moore et al., 2014). Dysregulation of AFF3 has been associated with various diseases such as rheumatoid arthritis (Stahl et al., 2010) and breast cancer (To et al., 2005).

A role for AFF3 in neural development was implicated after the discovery of a folate sensitive fragile site (FSFS), encompassing a CGG repeat expansion called FRA2A, in the *AFF3* gene promoter (Anneren and Gustavson, 1981; Murthy et al., 1990; Tukun et al., 2000). Hypermethylation of CGG repeats in these FSFS results in silencing of the surrounding locus, which is often associated with intellectual disability (Debacker and Kooy, 2007). FRA2A hypermethylation indeed leads to *AFF3* silencing and is associated with impaired motor and language skills (Metsu et al., 2014).

To date, AFF3 deletions (2) and missense (16) variants have been identified in 18 individuals with developmental delay and intellectual disability (Steichen-Gersdorf et al., 2008; Shimizu et al., 2019; Voisin et al., 2021). Interestingly, all missense variants located to the ALF domain of AFF3, similar to AFF4 missense variants (Raible et al., 2019). However, in contrast to the reported AFF4 variants, AFF3 protein stability was not affected (Voisin et al., 2021). Together with the observed gene deletions, this suggests that AFF3 heterozygous LoF causes the observed phenotype.

Besides developmental delay and intellectual disability, most patients carrying AFF3 variants presented with encephalopathy, skeletal dysplasia, failure to thrive, microcephaly and global brain atrophy (Steichen-Gersdorf et al., 2008; Shimizu et al., 2019; Voisin et al., 2021). Despite similarity to CHOPS, specific characteristics suggested a novel syndrome called KINSSHIP for horseshoe kidney, Nievergelt/Savarirayan type of mesomelic dysplasia, seizures, hypertrichosis, intellectual disability, and pulmonary involvement (OMIM# 619297) (Voisin et al., 2021). AFF3 missense variants or deletions cause a much more severe phenotype than FRA2A-associated *AFF3* gene silencing. This could be explained by a lack of AFF3 inactivation in the first few weeks after fertilization (Willemsen et al., 2002) or, alternatively, AFF3 silencing could be tissue specific (Voisin et al., 2021).

In mice, *Aff3* is expressed in cortical neurons during the initial steps of differentiation and is downregulated in the postnatal cortex (Britanova et al., 2002). Similarly, in humans, *AFF3* is highly expressed in the fetal brain and diminished in adults (Hiwatari et al., 2003). *Aff3* homo- and heterozygous knockout causes skeletal defect, and homozygous knockouts also show abnormal skull shape, kidney defects, brain malformations and neurological anomalies, similar to features presented in KINSSHIP probands (Voisin et al., 2021). Aff3 depletion in the developing mouse cortex resulted in neuronal migration defects that may explain the developmental delay and ID identified in humans with AFF3 haploinsufficiency (Moore et al., 2014).

### AFF2/FMR2

AF4/FRM2 family member 2 (*AFF2*), often referred to as *FMR2*, is an X-linked gene and known to encode a transcription activator (Hillman and Gecz, 2001). In contrast to the other AFF family members, AFF2 is not associated with ALL fusion. In the SEC, it functions as scaffolding protein by binding ENL or AF9 and P-TEFb to form SEC-like 2, which regulates a specific subset of genes (Luo et al., 2012).

Like AFF3, AFF2 also contains an FSFS site, located in the 5’UTR (untranslated region) of exon 1. CGG repeat expansion at this site leads to AFF2 silencing and can result in FRAXE intellectual disability (Gecz et al., 1996; Gu et al., 1996). FRAXE ID can be mild to severe, and include cognitive impairment, delayed language development, autistic behavior, and characteristics such as a long, narrow face, mild facial hypoplasia, a high-arched palate, irregular teeth, hair abnormality, angiomata, clinodactyly, thick lips, and nasal abnormalities (Flynn et al., 1993; Knight et al., 1993; Gecz et al., 1996; Gu et al., 1996).

Intragenic variants and chromosomal disruption of *AFF2* can also cause a similar phenotype (Gedeon et al., 1995; Honda et al., 2007; Sahoo et al., 2011; Stettner et al., 2011), indicating that it is indeed the hemizygous loss of AFF2 that leads to ID. Deletions always encompass the highly conserved ALF domain that is also affected in AFF3 and AFF4 LoF variants, underscoring its functional importance in relation to the ID phenotype. Dysregulation or other missense mutations are also associated with epilepsy (Timms et al., 1997; Moore et al., 1999) and ASD (Mondal et al., 2012).

*Aff2* is expressed in the subventricular zone (SVZ) and cortical plate of the mouse cortex (Chakrabarti et al., 1998; Gu and Nelson, 2003; Vogel and Gruss, 2009). *Aff2* knockout mice show impaired learning and memory performance and increased long-term potentiation in the hippocampus (Gu et al., 2002). Furthermore, *AFF2*-null neurons show reduced synaptic activity (Deneault et al., 2018) and silencing of *AFF2* in patients leads to deregulation of IEGs previously implicated in neuronal migration and activation, such as *JUN* and *FOS* (Perez-Cadahia et al., 2011; Bjorkblom et al., 2012; Melko et al., 2013). Dysregulation of *JUN* and *FOS* has been implicated in other ID disorders (e.g., related to Mediator complex mutations) (Hashimoto et al., 2011), suggesting they may be important downstream effector genes.

### AF9/MLLT3

*AF9*, also known as *MLLT3*, is one of the most common fusion partners of the *MLL* gene, and is often associated with leukemia (Strissel et al., 2000). Upon interaction with histone methyltransferase Dot1L, AF9 acts as an epigenetic modifier at specific genes to cause both activation and repression (Zhang et al., 2006; Bitoun et al., 2007; Buttner et al., 2010). A general role in transcription pause regulation was found for AF9, and its homolog ENL, through its contribution to the SEC (like) complexes (Lin et al., 2010; He et al., 2011). Here, the specific YEATS domains of AF9 and ENL interact with the PAF complex, resulting in recruitment of SEC to paused RNApol2 (He et al., 2011). Upon loss of AF9 and ENL, P-TEFb recruitment to NELF-A and the RNApol2 CTD is disrupted, inhibiting its phosphorylation and thereby pause release (Lu et al., 2016).

In two patients, *de novo* translocations of chromosome 4 and 9, *t*(4;9) were identified to cause a disruption of the *AF9* gene, resulting in neurodevelopmental delay with intellectual disability, growth delay, seizures and ataxia (Pramparo et al., 2005; Striano et al., 2005). Long-read sequencing in a patient with intellectual disability and facial dysmorphism rendered AF9 heterozygous LoF as likely causative gene (Hiatt et al., 2021).

*Af9* knockout mice display perinatal lethality (Collins et al., 2002). *Af9* is expressed in various brain regions, including the cortex, the hippocampus, cerebellar cortex and at the midbrain/hindbrain boundary (Vogel and Gruss, 2009). In the developing cortex, *Af9* expression prevents premature differentiation of TBR2-positive intermediate progenitor cells (IPCs) in the subventricular zone (SVZ) (Buttner et al., 2010). In human ESCs, AF9 interacts with the 5-methylcytosine dioxygenase TET2 to activate neural targets and support neural commitment (Qiao et al., 2015).

Taken together, these studies indicate that various SEC components have an important role in neural development, albeit not exclusively in the context of the SEC. Comparing patient phenotypes and commonly deregulated pathways should reveal the contribution of pausing dysregulation to the neurological symptoms.

## Integrator Complex

The Integrator complex is a > 1 MDa protein complex that is conserved across metazoans and consists of 14 subunits (Baillat et al., 2005; Malovannaya et al., 2011; Chen et al., 2012). It can directly bind the Ser7-phosphorylated RNApol2 CTD and, through catalytic subunit INTS11, mediate endonucleolytic 3′-end cleavage of many nascent RNAs (Egloff et al., 2010; Baillat and Wagner, 2015). Initially described as required for termination of small-nuclear RNA (snRNA) transcription, Integrator is now known to control processing and expression of other non-polyadenylated RNApol2 transcripts, including enhancer RNAs (eRNAs) (Lai et al., 2015; Elrod et al., 2019), telomerase RNAs (Rubtsova et al., 2019), viral miRNAs (Cazalla et al., 2011), replication-dependent histones, and long non-coding RNAs (Skaar et al., 2015).

A role for Integrator complex in the regulation of protein coding genes was first described in 2014, when two independent groups demonstrated its association with paused RNApol2. Integrator was shown to be required for initiation and pause-release of EGF-responsive IEGs in HeLa cells, where it interacts with NELF and is required for recruitment of the SEC (Gardini et al., 2014; Stadelmayer et al., 2014). Contrary to this stimulatory role in pause-release, Integrator complex has also been described as attenuator of transcription impinging on paused RNApol2. It stimulates premature termination through endonucleolytic cleavage of nascent RNA associated with the pausing complex (Elrod et al., 2019; Tatomer et al., 2019). Indeed, INTS9 or INTS11 depletion mainly resulted in upregulation of Integrator-bound genes both in *Drosophila* and human cells. Furthermore, association with protein phosphatase 2A (PP2A) provides Integrator with an alternative catalytic function to dampen transcriptional output through dephosphorylation of the RNApol2 CTD and DSIF-subunit SPT5 (Huang et al., 2020; Zheng et al., 2020; Vervoort et al., 2021).

Biallelic mutations in Integrator complex Subunits INTS8 have been identified in three siblings that manifest with a rare and recessive neurodevelopmental syndrome (Oegema et al., 2017). Features include severe intellectual disability, seizures, impaired speech development, motor impairment, facial dysmorphism and limb anomalies. Brain MRI scans showed microcephaly and structural brain abnormalities such as cerebellar hypoplasia, reduced volume of the pons and brainstem, and periventricular heterotopia, a cortical neuron migration defect (Table 1). The compound heterozygous INTS8 variants encompass a predicted missense mutation (c.893A > G, p.Asp298Gly) leading to an unstable transcript, and a nine-base-pair in-frame deletion leading to the deletion of three amino acids (c.2917_2925del, p.Glu972_Leu974del, or ‘ΔEVL’) (Oegema et al., 2017). INTS8-ΔEVL showed reduced association with the Integrator complex and RNApol2, leading to instability of other subunits and an overall loss of complex integrity. This resulted in misprocessing of UsnRNA, splicing defects and gene expression changes affecting neuronal differentiation (Oegema et al., 2017). In a separate study, INTS8 was shown to be required for association of PP2A with the Integrator complex (Huang et al., 2020). INTS8 depletion resulted in increased RNApol2 CTD and SPT5 phosphorylation, stimulating pause-release and thereby upregulation of Integrator target genes.

To date, ten patients carrying biallelic *INTS1* mutations have been reported in literature. They include homozygous missense (Krall et al., 2019) or non-sense variants (Oegema et al., 2017), as well as a combination of missense and either frameshift or non-sense variants (Krall et al., 2019; Zhang et al., 2020). All reported patients presented with growth and cognitive delay, severe language impairment, facial dysmorphism and cataracts. Skeletal malformations, in particular of the chest wall, and motor impairment were also frequently noted. Mechanistically it is not clear how INTS1 variants impact on Integrator complex function, although its potential role as scaffolding subunit could affect the function of the entire complex. In addition, subunit cross-regulation has been reported in zebrafish models, where ints1 depletion had a negative impact on expression of other Integrator subunits (Krall et al., 2019).

Depletion of Ints1 and Ints11 from neural progenitors in the developing mouse brain resulted in neuronal migration defects, linked to aberrant semaphorin signaling (van den Berg et al., 2017). Similar defects were observed upon disruption of NIPBL, a novel interactor of the Integrator complex and prominent causal factor in CdLS. Interestingly, multiple overlapping clinical features between CdLS and INTS mutations have been reported in literature, which are summarized in Table 1. Besides growth and cognitive delays, common facial abnormalities (e.g., micrognathia, downturned corners of the mouth, widely spaced teeth), pectus deformity and renal malformations were frequently reported (Kline et al., 2018; Krall et al., 2019). In line with the data from mouse neural progenitor cells, this indeed suggests dysregulation of common gene regulatory pathways as underlying cause of the observed clinical features.

## ARID1A/ARID1B

AT-rich interactive domain-containing protein 1A (ARID1A) and 1B (ARID1B) are one of the main, mutually exclusive subunits of the switch/sucrose non-fermentable (SWI/SNF)-like brahma-associated factor (BAF) complex, a multiprotein ATP-dependent chromatin remodeling complex composed of conserved core- and variant subunits (Raab et al., 2015; Mashtalir et al., 2018). SWI/SNF complexes play important roles in epigenetic regulation of gene expression, lineage specification, and maintenance of stem cell pluripotency (Euskirchen et al., 2011; Raab et al., 2015). ARID1A-containing complexes are particularly involved in tumor suppression, and ARID1A is the most frequently mutated chromatin regulator across all human cancers (Lawrence et al., 2014). In particular, ovarian clear cell carcinoma (OCCC) carries the highest prevalence of ARID1A mutations (∼57%) (Jones et al., 2010).

Mutations in *ARID1A* and *ARID1B* are also an important cause of Coffin-Siris Syndrome (CSS; OMIM 135900), a rare autosomal-dominant neurodevelopmental syndrome (Tsurusaki et al., 2012). CSS is characterized by intellectual disability, growth deficiency, microcephaly, coarse facial features and hypoplastic or absent nail of the fifth finger or toe (Coffin and Siris, 1970). Approximately 60% of affected individuals carry a germline mutation in one of six SWI/SNF subunit genes (SMARCB1, SMARCA4, SMARCA2, SMARCE1, ARID1A, and ARID1B) or a small set of additional genes (Vergano et al., 1993; Santen et al., 2012, 2013; Tsurusaki et al., 2012, 2014; Wieczorek et al., 2013). ARID mutations are mostly truncating, LoF mutations or whole-gene deletions, suggesting that haploinsufficiency is the likely cause of the observed neurodevelopmental phenotype (Bögershausen and Wollnik, 2018). Mostly heterozygous LoF mutations in ARID1A have been identified in around 5% of classic CSS cases (Vergano et al., 1993; Tsurusaki et al., 2012), whilst four CSS-like patients with *ARID1A* microduplications have been described (Bidart et al., 2017). *ARID1B* mutations have been found in up to 62% of (often milder) CSS cases and also explain a significant fraction (0,4–1,0%) of idiopathic ID cases that are often accompanied by speech impairment and agenesis of the corpus callosum hallmarks (Santen et al., 2012; Tsurusaki et al., 2012; Wieczorek et al., 2013; Grozeva et al., 2015).

Several studies have addressed the contribution of ARID1A and ARID1B in SWI/SNF-mediated gene regulation. These concluded that ARID1A-containing BAF acts as both transcriptional activator and repressor, whereas ARID1B-BAF mainly functions as repressor of enhancer activity (Raab et al., 2015). In addition, ARID1A and ARID1B were required for maintenance of global chromatin accessibility (Kelso et al., 2017) and, in the case of ARID1A, for genome compartmentalization (Wu et al., 2019). Interestingly, Trizzino et al. (2018) recently provided evidence for a role of ARID1A and ARID1B in transcription pause release. Knockdown of ARID1A reduced RNApol2 pausing on active genes and globally diminished Ser5-phosphorylation of the RNApol2 CTD. The pausing defect could be rescued by upregulation of ARID1B, suggesting that both ARID1A and ARID1B control transcription *via* RNApol2 pausing and that dysregulated pausing likely mediates effects of ARID1A loss in cancers and possibly also neurodevelopmental disorders.

Several studies have addressed the role of Arid1a and Arid1b in the developing mouse brain. Cortex-specific homozygous deletion of *Arid1a* resulted in reduced cortical thickness linked to inhibition of IPC proliferation and decreased production of deep layer neurons (Liu et al., 2021). In contrast, Arid1b deletion mainly affected ventral forebrain progenitors, suggesting differential requirement for Arid1a and Arid1b in distinct cellular compartments (Moffat et al., 2021). Indeed, CSS-mimicking *Arid1b*-heterozygous mice mainly showed defects in interneuron development (Jung et al., 2017). Reduced proliferation and increased apoptosis in progenitors of the lateral and medial ganglionic eminences (LGE and MGE) resulted in an overall decrease in GABA^+^ and Parvalbumin^+^ interneurons in the cortex. These developmental defects resulted in CSS-reminiscent behavioral abnormalities, including impaired cognitive function and social interaction, and increased anxiety-like and repetitive behavior (Celen et al., 2017; Jung et al., 2017). Interestingly, heterozygous Arid1b loss caused a strong reduction in Ser5-CTD phosphorylated RNApol2 at target gene promoters (Jung et al., 2017), warranting further investigation into the role of transcriptional pausing misregulation in CSS.

## PAF1 Complex

The Polymerase-Associated Factor 1 complex (PAF1c) is a multifunctional and highly conserved protein complex that regulates all stages of the RNA transcription cycle [recently reviewed in Francette et al. (2021)]. PAF1c was discovered as a novel RNApol2-interacting complex in *Saccharomyces cerevisiae* 25 years ago (Wade et al., 1996), and foundational studies in budding yeast have elucidated the diverse ways *via* which it controls gene expression (Wade et al., 1996; Shi et al., 1997; Mueller and Jaehning, 2002). PAF1c is composed of subunits PAF1, CTR9, CDC73, LEO1, RTF1 and, in human cells, SKI8 (Mueller and Jaehning, 2002). In higher eukaryotes, PAF1c is recruited to promoters and enhancers of active genes, where it directly binds to the CTD and outer surface of RNApol2, as well as to elongation factor SPT4/5 (DSIF) (Yu et al., 2015; Chen et al., 2021).

In recent years, the role of PAF1c in regulation of RNApol2 pause-release has been studied in detail. PAF1c strongly associates with P-TEFb and both factors show interdependent recruitment to target gene promoters (Yu et al., 2015). RNAi-mediated PAF1c depletion, depending on the cell line and specific study, resulted in either increased or decreased RNApol2 pausing (Yu et al., 2015; Chen et al., 2021). In both cases, observed effects were linked to alterations in P-TEFb recruitment. Interestingly, in zebrafish neural crest (NC) progenitors, loss of Paf1c could be compensated by loss of Cdk9, suggesting that at crucial NC genes Paf1c and P-TEFb act antagonistically (Jurynec et al., 2019). These contrasting findings may indicate that PAF1c function is context- and gene-specific. Detailed structural studies of the activated RNApol2 elongation complex showed that PAF1c displaces NELF, suggesting it mainly acts to promote pause-release (Vos et al., 2018a).

Pathogenic variants in genes encoding subunits or interactors of PAF1c have been identified in various neurodevelopmental processes and ID disorders. Most well-described are mutations in the X-linked gene encoding the PHD-Like Zinc Finger Protein 6 (PHF6) that associates with PAF1c and causes the ID disorder Börjeson–Forssman–Lehmann syndrome (BFLS; OMIM 301900) (Lower et al., 2002; Zhang et al., 2013). Besides ID, BFLS is characterized by epilepsy, hypometabolism, hypogonadism, obesity with gynecomastia, swollen subcutaneous facial tissues, narrow palpebral fissure, and large ears (Table 1) (Lower et al., 2002; Jahani-Asl et al., 2016). Eight different mutations, including two truncating non-sense variants and 6 different missense variants, were identified in seven familial and two sporadic cases of BFLS.

*Phf6* is highly expressed in embryonic and early postnatal stages of mouse brain development (Lower et al., 2002). RNAi-mediated Phf6 depletion *via in utero* electroporation of the embryonic mouse brain was shown to profoundly impair neuronal migration *in vivo*, leading to formation of white matter heterotopias that displayed neuronal hyperexcitability (Zhang et al., 2013). Paf1 depletion phenocopied this migration phenotype, suggesting important PHF6-PAF1c co-operation in transcription regulation of neurodevelopment.

PAF1c core subunit LEO1 was identified as candidate neurodevelopmental disease gene in a large meta-analysis study that combined ID and ASD patient *de novo* mutations with CNV morbidity data (Coe et al., 2019). This finding was confirmed in a targeted sequencing study 1 year later, where LEO1 mutations were linked to intellectual disability and autistic behavior (Wang et al., 2020). Paternally inherited deletions in the LEO1 promoter linked to increased LEO1 expression were also associated with ASD (Brandler et al., 2018), suggesting that PAF1c subunit imbalance may contribute to the neurodevelopmental phenotype. To date, PAF1c subunit CTR9 has not been causally linked to NDDs in humans, despite a clear neurological phenotype in *Drosophila*. In this model organism, embryonic or early larval lethality of Ctr9 mutants could be partially rescued by re-expression of Ctr9 in the nervous system and mutant embryos contained increased numbers of neuroblasts and dividing progeny (Bahrampour and Thor, 2016). Moreover, a role for Ctr9 in controlling terminal neuronal differentiation was proposed, as evidenced by downregulation of several neuropeptides.

Of note, besides PHF6, several additional PAF1c interactors have been implicated in pausing regulation and NDD, most prominently SETD5 (Osipovich et al., 2016) and CHD1 (Lee et al., 2017). The ATP-dependent chromatin remodeller CHD1 is recruited to actively transcribed genes by PAF1c (Lee et al., 2017), where it enables RNApol2 promoter escape by removing the nucleosome barrier (Skene et al., 2014). Heterozygous *CHD1* missense variants have been identified as the cause of developmental delay, autism, speech apraxia and facial dysmorphic features in Pilarowski–Bjornsson syndrome (OMIM #602118) (Pilarowski et al., 2018). SETD5 harbors H3K36 methyltransferase activity and is important for PAF1c recruitment to common target genes (Sessa et al., 2019; Li et al., 2021b). Setd5 depletion from hematopoietic stem cells resulted in decreased pausing indices, with a concomitant increase in elongating RNApol2 and upregulated target gene expression (Li et al., 2021b). *SETD5* heterozygous LoF variants are a leading cause of idiopathic ID and ASD (Kuechler et al., 2015; Deliu et al., 2018; Powis et al., 2018) and have also been linked to a CdLS-like phenotype (Parenti et al., 2017). Setd5 haploinsufficient mice show cognitive impairment and behavioral abnormalities linked to increased progenitor proliferation and a loss of synaptic contacts (Deliu et al., 2018; Sessa et al., 2019; Nakagawa et al., 2020).

## Mediator Complex

The Mediator complex interacts with different TFs and is implicated in almost every aspect of transcription regulation [reviewed by Jeronimo and Robert (2017)], chromatin architecture [reviewed by Andre et al. (2021)], and DNA repair (Soutourina and Werner, 2014). Due to its many functions, Mediator is often associated with cancer and developmental disease (Schiano et al., 2014; Yin and Wang, 2014). Mediator is composed of 30 subunits, organized in four parts: head, middle, tail and kinase. Each of these modules contains a specific set of Mediator subunits, and the specific composition of Mediator varies. The head and middle part interact with RNApol2 and general TFs at promoter sites, while the tail interacts with sequence specific TFs at enhancer sites (Tsai et al., 2014, 2017; Robinson et al., 2016). It is therefore hypothesized that Mediator, together with cohesin, facilitates chromatin looping to allow proximity of enhancers and promoters and enable PIC assembly (Kagey et al., 2010; Soutourina, 2018). In addition, Mediator, together with TFs, BRD4 and RNApol2, enables liquid-phase separation to form condensates of transcriptional machinery at super enhancers (Cho et al., 2018; Sabari et al., 2018).

Mediator was also found to regulated pausing in various ways. *In vitro*, Mediator overcomes the inhibitory activity of Gdown1 (Hu et al., 2006; Jishage et al., 2012), a stabilizer of promoter-proximal pausing, suggesting that Mediator might alleviate Gdown1-mediated blocking of paused RNApol2 (Cheng et al., 2012). Moreover, loss of MED14, which forms the interaction point between head and middle modules of Mediator, results in loss of promoter proximal RNApol2 (Jaeger et al., 2020). Mediator also regulates transcription pausing through interaction with pausing factors. Various Mediator subunits are found to directly interact with SEC, BRD4 and DSIF. Metazoan specific MED26 interacts with the EAF subunit in SEC, allowing recruitment of active P-TEFb to the pausing complex (Lu et al., 2016). Consequently, depletion of MED26 interrupts SEC recruitment, RNApol2 CTD phosphorylation and expression of *c-MYC* and *HSP70* genes (Takahashi et al., 2011). Furthermore, MED1 and MED23 recruit active P-TEFb associated with BRD4 to paused RNApol2 (Lu et al., 2016). In line with these findings, *Med23* knockout mESCs showed decreased P-TEFb binding to selected genes (Wang et al., 2013).

The kinase module associates with Mediator in a reversible manner and is best described out of all Mediator modules. It comprises of CDK8, CCNC, MED12, and MED13. In vertebrates, paralogs of CDK8, MED12, and MED13 are CDK19, MED12L, and MED13L, respectively. Although the exact function of these paralogs is unknown, they incorporate into the Mediator complex in a mutually exclusive manner (Daniels et al., 2013). CDK8 depletion leads to reduction of SEC recruitment to the promoter site of serum induced genes, resulting in reduced gene expression (Donner et al., 2010). Similarly, SEC is recruited to hypoxia-inducible genes in a CDK8 dependent manner (Galbraith et al., 2013), suggesting a role for Mediator’s kinase module in transcription pause regulation.

### Kinase Module

Variants in all proteins of the kinase module, except for CCNC, have been implicated in neurodevelopmental delay, indicating an important role for this module in neuronal development. A total of fourteen patients have been described with 10 different *de novo* heterozygous missense mutations in CDK8 (Calpena et al., 2019; Uehara et al., 2020). These patients suffer from hypotonia, ID and variable facial dysmorphisms, as well as agenesis of the corpus callosum. For CDK19, 15 patients were identified that presented with a similar phenotype (Mukhopadhyay et al., 2010; Chung et al., 2020; Zarate et al., 2021), suggesting that both kinases perform similar, non-redundant functions. Besides one case of CDK19 haploinsufficiency, all disease-causing missense variants for CDK8 and CDK19 are localized in the kinase domain (Chung et al., 2020; Zarate et al., 2021), indicating that loss of kinase activity contributes to the neurological defects that involve reduced dendritic branching and altered dendrite morphology (Mukhopadhyay et al., 2010).

From all kinase module subunits, variants in the X-linked gene *MED12* have most frequently been identified in NDDs. Hemizygous variants in males have been described to cause Opitz–Kaveggia syndrome (OMIM# 305450), Ohdo syndrome (OMIM# 300895), and Lujan–Fryns syndrome (OMIM# 309520) (Risheg et al., 2007; Schwartz et al., 2007; Vulto-van Silfhout et al., 2013). These three syndromes are very similar, as they all encompass ID, macrocephaly, hypotonia, abnormalities in the corpus callosum and typical facial features. However, most patients with MED12 variants, including a total of 25 females (Li et al., 2021a; Polla et al., 2021), show syndromic or non-syndromic ID without a specific disease phenotype [reviewed in Plassche and Brouwer (2021) and Srivastava and Kulshreshtha (2021)]. Non-sense, missense, and splice-site variants localize to all protein domains, resulting in a large spectrum of phenotypes with varying severities of ID and developmental delay. Recently, seven individuals were identified with mutations in MED12L, a MED12 paralog (Nizon et al., 2019). These encompass a wide variety of heterozygous mutations, such as duplication, deletion or single-nucleotide variants. Patients present with ID, developmental delay, speech impairment, and sometimes abnormalities in the corpus callosum.

Although the exact mechanism by which MED12 causes this neuronal phenotype remains to be elucidated, several relevant pathways have been described. For example, MED12 interacts with G9a and REST to regulate neuronal gene expression (Ding et al., 2008, 2009). Moreover, MED12 interacts with Gli3 to activate SHH target genes (Zhou et al., 2006) and some of the reported MED12 missense variants were unable SHH target gene expression in *Med12* null mice (Zhou et al., 2012). Importantly, several MED12 variants affected expression of IEGs such as *JUN*, *FOS* and *EGR1*, which is controlled at the level of pause-release (Donnio et al., 2017).

MED13 and in particular MED13L variants have been linked to various neurodevelopmental aberrations. MED13 was first described as candidate ID gene in a patient with short stature and mild dysmorphisms caused by an 800 kb deletion (Boutry-Kryza et al., 2012). MED13 variants were subsequently described in patients with ASD (Iossifov et al., 2014; Rk et al., 2017) and, recently, missense or truncating variants were identified in thirteen patients presenting with developmental delay, ID, and speech disorders (Snijders Blok et al., 2018). MED13L patients show developmental delay and ID and many of the phenotypes also observed for MED13 and MED12 variants, such as ASD and hypotonia (Asadollahi et al., 2013; Adegbola et al., 2015; Cafiero et al., 2015; van Haelst et al., 2015; Torring et al., 2019; Plassche and Brouwer, 2021). Most frequently, this is caused by a heterozygous loss-of-function of MED13L, and the most severe phenotypes seem to be caused by missense mutations, indicative of a dominant-negative effect (Smol et al., 2018). However, the exact mechanism and pathways remain unknown. In a recent mouse study, Hamada et al. (2021) showed that MED13L protein is highly expressed in the ventricular zone of the cerebral cortex and is also detectable in the developing hippocampus and cerebellum.

### Tail and Head Module

Although proteins of the kinase module are most often described in connection with neuronal development, Mediator components MED17 (head), MED23, MED25, and MED27 (tail) have also been found to cause neurodevelopmental disorders upon mutation. In seven individuals, biallelic mutations of MED23 were identified to cause ID (Hashimoto et al., 2011; Trehan et al., 2015). A homozygous variant found in five of these patients was shown to have altered interaction with enhancer bound TFs, resulting in dysregulation of IEGs *JUN* and *FOS* (Hashimoto et al., 2011).

The first disease causing variant identified in MED25, a homozygous missense mutation in the SH3 recognition domain, was detected in a consanguineous family in which 23 individuals presented with Charcot-Marie-Tooth disease type 2B2, a peripheral axonal neuropathy (Leal et al., 2009). Two other studies identified a total of 14 individuals with moderate to severe ID, who were also affected by two different homozygous *MED25* missense mutations (Basel-Vanagaite et al., 2015; Figueiredo et al., 2015). In one study patients also presented with abnormalities in the eye, palate and corpus callosum (Basel-Vanagaite et al., 2015). The missense mutation found in this study drastically impairs MED25 interaction with other Mediator components.

Interestingly, autosomal recessive variants in MED27 and MED17 result in a very similar disease phenotype, characterized by developmental delay, spasticity, seizures, microcephaly and cerebellar atrophy (Kaufmann et al., 2010; Meng et al., 2021). Metazoan-specific subunit MED27 forms the junction between the head and tail modules of the Mediator complex, where it interacts with MED14 and MED17 (Rengachari et al., 2021). Disrupted interaction between head and tail modules might therefore contribute to the neurodevelopmental abnormalities. Indeed, in zebrafish, Med27 loss of function disrupts dopaminergic amacrine cells and serotonergic neurons resulting in size reduction of head, eye, jaw, and eventually leading to lethality 6 days post fertilization (Durr et al., 2006). In fly and chicken, disruption of Med27 also leads to embryonic lethality (Gokcezade et al., 2014; Li-Kroeger et al., 2018; Tsujino et al., 2019). Together these results clearly indicate an important role for Med27 in embryonic and neuronal development.

Taken together, it can be concluded that many of the Mediator components are already found to be essential for neuronal development and, when mutated, can cause syndromic ID in various forms. Despite phenotypic variety, intellectual disability arises in almost all cases. Interestingly, many NDD-causing mutations converge on the kinase module, which has been implicated in the regulation of transcriptional pause-release (Donner et al., 2010; Galbraith et al., 2013; Poot, 2020). Therefore, it would be of particular interest to further delineate the molecular and developmental defects downstream of these mutations.

## Conclusion

Transcription pause-release is increasingly being recognized as an important step in the regulation of gene expression. Here we have highlighted several neurodevelopmental disorders that are likely caused by dysregulated transcriptional pausing. To what extent the reported variants affect pause duration in the developing brain and which genes and pathways are most affected should be subject of future studies. Several experimental approaches can be used to measure pause duration. For example, RNApol2 positional information obtained from relatively small cell populations using CUT&RUN-based methods (Meers et al., 2019) can be used to calculate gene-specific pausing indices. Furthermore, nascent RNA-sequencing techniques such as precision nuclear run-on (PRO) sequencing (Kwak et al., 2013) or mammalian native elongating transcript sequencing (mNET-seq) (Nojima et al., 2015) map active RNApol2 with (near-) nucleotide resolution in a highly quantitative manner. Recent adaptations (Judd et al., 2020) mean that it will become feasible to apply these techniques to small, pure cell populations isolated from developing human brain organoids, thereby potentially enabling the establishment of a direct link between transcriptional pausing and neurodevelopmental defects.

Shared clinical features and diagnosis (notably the frequent occurrence of CdLS-like characteristics) suggest a common underlying mechanism. In this respect it will be interesting to investigate a possible role in the regulation of transcription pause-release for additional factors that, when mutated, result in a CdLS-like appearance (i.e., KMT2A and ANKRD11). Indeed, a link between KMT2A and transcriptional pausing has been suggested in HIV latency reversal, where KMT2A displaces PAF1c and facilitates SEC recruitment (Gao et al., 2020). Following the reverse logic, variants in known pausing regulators [e.g., TRIM28 (Bunch et al., 2014)] should be given special consideration during genetic diagnosis. Furthermore, gene editing techniques and advanced *in vitro* models of human brain development (e.g., brain organoids) now provide an excellent opportunity to uncover the disease-relevant neurodevelopmental pathways typically affected by dysregulated pause-release. We envision that this knowledge can directly translate into improved diagnostics in the clinic, by providing evidence for gene variant causality and through transcriptome-based diagnostics. Finally, as transcription pause-release is a process amenable to drug intervention, it forms a potentially promising target for drug-mediated therapeutic intervention in NDDs.

## Author Contributions

All authors listed have made a substantial, direct, and intellectual contribution to the work, and approved it for publication.

## Conflict of Interest

The authors declare that the research was conducted in the absence of any commercial or financial relationships that could be construed as a potential conflict of interest.

## Publisher’s Note

All claims expressed in this article are solely those of the authors and do not necessarily represent those of their affiliated organizations, or those of the publisher, the editors and the reviewers. Any product that may be evaluated in this article, or claim that may be made by its manufacturer, is not guaranteed or endorsed by the publisher.

## References

[B1] Adegbola A. Musante L. Callewaert B. Maciel P. Hu H. Isidor B. (2015). Redefining the MED13L syndrome. *Eur. J. Hum. Genet.* 23 1308–1317. 10.1038/ejhg.2015.26 25758992 PMC4592099

[B2] Adelman K. Lis J. T. (2012). Promoter-proximal pausing of RNA polymerase II: emerging roles in metazoans. *Nat. Rev. Genet.* 13 720–731. 10.1038/nrg3293 22986266 PMC3552498

[B3] Alazami A. M. Patel N. Shamseldin H. E. Anazi S. Al-Dosari M. S. Alzahrani F. (2015). Accelerating novel candidate gene discovery in neurogenetic disorders *via* whole-exome sequencing of prescreened multiplex consanguineous families. *Cell Rep.* 10 148–161. 10.1016/j.celrep.2014.12.015 25558065

[B4] Alesi V. Dentici M. L. Loddo S. Genovese S. Orlando V. Calacci C. (2019). Confirmation of BRD4 haploinsufficiency role in Cornelia de Lange-like phenotype and delineation of a 19p13.12p13.11 gene contiguous syndrome. *Ann. Hum. Genet.* 83 100–109. 10.1111/ahg.12289 30302754

[B5] Andre K. M. Sipos E. H. Soutourina J. (2021). Mediator roles going beyond transcription. *Trends Genet.* 37 224–234. 10.1016/j.tig.2020.08.015 32921511

[B6] Aneichyk T. Hendriks W. T. Yadav R. Shin D. Gao D. Vaine C. A. (2018). Dissecting the causal mechanism of x-linked dystonia-parkinsonism by integrating genome and transcriptome assembly. *Cell* 172 897–909.e21. 10.1016/j.cell.2018.02.011 29474918 PMC5831509

[B7] Anneren G. Gustavson K. H. (1981). A fragile secondary constriction on chromosome 2 in five patients with different clinical features. *Hereditas* 95 63–67. 10.1111/j.1601-5223.1981.tb01329.x 7333874

[B8] Ansari M. Poke G. Ferry Q. Williamson K. Aldridge R. Meynert A. M. (2014). Genetic heterogeneity in Cornelia de Lange syndrome (CdLS) and CdLS-like phenotypes with observed and predicted levels of mosaicism. *J. Med. Genet.* 51 659–668. 10.1136/jmedgenet-2014-102573 25125236 PMC4173748

[B9] Aoi Y. Takahashi Y. H. Shah A. P. Iwanaszko M. Rendleman E. J. Khan N. H. (2021). SPT5 stabilization of promoter-proximal RNA polymerase II. *Mol Cell.* 28:778. 10.1016/j.molcel.2021.08.006 34480849 PMC8687145

[B10] Arnold M. Bressin A. Jasnovidova O. Meierhofer D. Mayer A. (2021). A BRD4-mediated elongation control point primes transcribing RNA polymerase II for 3’-processing and termination. *Mol. Cell.* 81 3589–3603.e13. 10.1016/j.molcel.2021.06.026 34324863

[B11] Asadollahi R. Oneda B. Sheth F. Azzarello-Burri S. Baldinger R. Joset P. (2013). Dosage changes of MED13L further delineate its role in congenital heart defects and intellectual disability. *Eur. J. Hum. Genet.* 21 1100–1104. 10.1038/ejhg.2013.17 23403903 PMC3778355

[B12] Bacon C. W. D’Orso I. (2019). CDK9: a signaling hub for transcriptional control. *Transcription* 10 57–75. 10.1080/21541264.2018.1523668 30227759 PMC6602564

[B13] Bahrampour S. Thor S. (2016). Ctr9, a key component of the Paf1 complex, affects proliferation and terminal differentiation in the developing drosophila nervous system. *G3 Genes| Genomes| Genetics* 6 3229–3239. 10.1534/g3.116.034231 27520958 PMC5068944

[B14] Baillat D. Wagner E. J. (2015). Integrator: surprisingly diverse functions in gene expression. *Trends Biochem. Sci.* 40 257–264. 10.1016/j.tibs.2015.03.005 25882383 PMC4408249

[B15] Baillat D. Hakimi M. A. Näär A. M. Shilatifard A. Cooch N. Shiekhattar R. (2005). Integrator, a multiprotein mediator of small nuclear RNA processing, associates with the C-terminal repeat of RNA polymerase II. *Cell* 123 265–276. 10.1016/j.cell.2005.08.019 16239144

[B16] Barboric M. Kohoutek J. Price J. P. Blazek D. Price D. H. Peterlin B. M. (2005). Interplay between 7SK snRNA and oppositely charged regions in HEXIM1 direct the inhibition of P-TEFb. *EMBO J.* 24 4291–4303. 10.1038/sj.emboj.7600883 16362050 PMC1356324

[B17] Bartman C. R. Hamagami N. Keller C. A. Giardine B. Hardison R. C. Blobel G. A. (2019). Transcriptional burst initiation and polymerase pause release are key control points of transcriptional regulation. *Mol. Cell.* 73 519–532.e4. 10.1016/j.molcel.2018.11.004 30554946 PMC6368450

[B18] Basel-Vanagaite L. Smirin-Yosef P. Essakow J. L. Tzur S. Lagovsky I. Maya I. (2015). Homozygous MED25 mutation implicated in eye-intellectual disability syndrome. *Hum. Genet.* 134 577–587. 10.1007/s00439-015-1541-x 25792360

[B19] Bidart M. El Atifi M. Miladi S. Rendu J. Satre V. Ray P. F. (2017). Microduplication of the ARID1A gene causes intellectual disability with recognizable syndromic features. *Genet. Med.* 19 701–710. 10.1038/gim.2016.180 27906199

[B20] Bisgrove D. A. Mahmoudi T. Henklein P. Verdin E. (2007). Conserved P-TEFb-interacting domain of BRD4 inhibits HIV transcription. *Proc. Natl. Acad. Sci. U S A.* 104 13690–13695. 10.1073/pnas.0705053104 17690245 PMC1959443

[B21] Biswas D. Milne T. A. Basrur V. Kim J. Elenitoba-Johnson K. S. Allis C. D. (2011). Function of leukemogenic mixed lineage leukemia 1 (MLL) fusion proteins through distinct partner protein complexes. *Proc. Natl. Acad. Sci. U S A.* 108 15751–15756. 10.1073/pnas.1111498108 21896721 PMC3179097

[B22] Bitoun E. Davies K. E. (2005). The robotic mouse: unravelling the function of AF4 in the cerebellum. *Cerebellum* 4 250–260. 10.1080/14734220500325897 16321881

[B23] Bitoun E. Oliver P. L. Davies K. E. (2007). The mixed-lineage leukemia fusion partner AF4 stimulates RNA polymerase II transcriptional elongation and mediates coordinated chromatin remodeling. *Hum. Mol. Genet.* 16 92–106. 10.1093/hmg/ddl444 17135274

[B24] Bjorkblom B. Padzik A. Mohammad H. Westerlund N. Komulainen E. Hollos P. (2012). c-Jun N-terminal kinase phosphorylation of MARCKSL1 determines actin stability and migration in neurons and in cancer cells. *Mol. Cell. Biol.* 32 3513–3526. 10.1128/MCB.00713-12 22751924 PMC3421996

[B25] Bögershausen N. Wollnik B. (2018). Mutational landscapes and phenotypic spectrum of SWI/SNF-Related intellectual disability disorders. *Front. Mol. Neurosci.* 11:252. 10.3389/fnmol.2018.00252 PMC6085491 30123105

[B26] Boija A. Klein I. A. Sabari B. R. Dall’agnese A. Coffey E. L. Zamudio A. V. (2018). Transcription factors activate genes through the phase-separation capacity of their activation domains. *Cell* 175 1842–1855.e16. 10.1016/j.cell.2018.10.042 30449618 PMC6295254

[B27] Bonaglia M. C. Marelli S. Novara F. Commodaro S. Borgatti R. Minardo G. (2010). Genotype-phenotype relationship in three cases with overlapping 19p13.12 microdeletions. *Eur. J. Hum. Genet.* 18 1302–1309. 10.1038/ejhg.2010.115 20648052 PMC3002847

[B28] Boutry-Kryza N. Labalme A. Till M. Schluth-Bolard C. Langue J. Turleau C. (2012). An 800 kb deletion at 17q23.2 including the MED13 (THRAP1) gene, revealed by aCGH in a patient with a SMC 17p. *Am. J. Med. Genet. A* 158A 400–405. 10.1002/ajmg.a.34222 22162340

[B29] Brandler W. M. Antaki D. Gujral M. Kleiber M. L. Whitney J. Maile M. S. (2018). Paternally inherited cis-regulatory structural variants are associated with autism. *Science* 360 327–331. 10.1126/science.aan2261 29674594 PMC6449150

[B30] Braunholz D. Obieglo C. Parenti I. Pozojevic J. Eckhold J. Reiz B. (2015). Hidden mutations in Cornelia de Lange syndrome limitations of sanger sequencing in molecular diagnostics. *Hum. Mutat.* 36 26–29. 10.1002/humu.22685 25196272

[B31] Britanova O. Lukyanov S. Gruss P. Tarabykin V. (2002). The mouse Laf4 gene: exon/intron organization, cDNA sequence, alternative splicing, and expression during central nervous system development. *Genomics* 80 31–37. 10.1006/geno.2002.6796 12079280

[B32] Brunkhorst A. Karlen M. Shi J. Mikolajczyk M. Nelson M. A. Metsis M. (2005). A specific role for the TFIID subunit TAF4 and RanBPM in neural progenitor differentiation. *Mol. Cell. Neurosci.* 29 250–258. 10.1016/j.mcn.2005.02.015 15911349

[B33] Bunch H. Zheng X. Burkholder A. Dillon S. T. Motola S. Birrane G. (2014). TRIM28 regulates RNA polymerase II promoter-proximal pausing and pause release. *Nat. Struct. Mol. Biol.* 21 876–883. 10.1038/nsmb.2878 25173174 PMC4189995

[B34] Busslinger G. A. Stocsits R. R. Van Der Lelij P. Axelsson E. Tedeschi A. Galjart N. (2017). Cohesin is positioned in mammalian genomes by transcription, CTCF and Wapl. *Nature* 544 503–507. 10.1038/nature22063 28424523 PMC6080695

[B35] Buttner N. Johnsen S. A. Kugler S. Vogel T. (2010). Af9/Mllt3 interferes with Tbr1 expression through epigenetic modification of histone H3K79 during development of the cerebral cortex. *Proc. Natl. Acad. Sci. U S A.* 107 7042–7047. 10.1073/pnas.0912041107 20348416 PMC2872432

[B36] Cafiero C. Marangi G. Orteschi D. Ali M. Asaro A. Ponzi E. (2015). Novel de novo heterozygous loss-of-function variants in MED13L and further delineation of the MED13L haploinsufficiency syndrome. *Eur. J. Hum. Genet.* 23 1499–1504. 10.1038/ejhg.2015.19 25712080 PMC4613466

[B37] Calpena E. Hervieu A. Kaserer T. Swagemakers S. M. A. Goos J. A. C. Popoola O. (2019). De novo missense substitutions in the gene encoding CDK8, a regulator of the mediator complex, cause a syndromic developmental disorder. *Am. J. Hum. Genet.* 104 709–720. 10.1016/j.ajhg.2019.02.006 30905399 PMC6451695

[B38] Capponi S. Stoffler N. Irimia M. Van Schaik F. M. A. Ondik M. M. Biniossek M. L. (2020). Neuronal-specific microexon splicing of TAF1 mRNA is directly regulated by SRRM4/nSR100. *RNA Biol.* 17 62–74. 10.1080/15476286.2019.1667214 31559909 PMC6948980

[B39] Castronovo P. Gervasini C. Cereda A. Masciadri M. Milani D. Russo S. (2009). Premature chromatid separation is not a useful diagnostic marker for Cornelia de Lange syndrome. *Chromosome Res.* 17 763–771. 10.1007/s10577-009-9066-6 19690971

[B40] Cazalla D. Xie M. Steitz J. A. (2011). A primate herpesvirus uses the integrator complex to generate viral microRNAs. *Mol. Cell.* 43 982–992. 10.1016/j.molcel.2011.07.025 21925386 PMC3176678

[B41] Celen C. Chuang J. C. Luo X. Nijem N. Walker A. K. Chen F. (2017). Arid1b haploinsufficient mice reveal neuropsychiatric phenotypes and reversible causes of growth impairment. *eLife* 6:e25730. 10.7554/eLife.25730 28695822 PMC5515576

[B42] Chakrabarti L. Bristulf J. Foss G. S. Davies K. E. (1998). Expression of the murine homologue of FMR2 in mouse brain and during development. *Hum. Mol. Genet.* 7 441–448. 10.1093/hmg/7.3.441 9467002

[B43] Chen F. X. Smith E. R. Shilatifard A. (2018). Born to run: control of transcription elongation by RNA polymerase II. *Nat. Rev. Mol. Cell Biol.* 19 464–478. 10.1038/s41580-018-0010-5 29740129

[B44] Chen F. Liu B. Zeng J. Guo L. Ge X. Feng W. (2021). Crystal structure of the core module of the yeast Paf1 complex. *J. Mol. Biol.* 434:167369. 10.1016/j.jmb.2021.167369 34852272

[B45] Chen J. Ezzeddine N. Waltenspiel B. Albrecht T. R. Warren W. D. Marzluff W. F. (2012). An RNAi screen identifies additional members of the *Drosophila* integrator complex and a requirement for cyclin C/Cdk8 in snRNA 3’-end formation. *RNA* 18 2148–2156. 10.1261/rna.035725.112 23097424 PMC3504667

[B46] Cheng B. Li T. Rahl P. B. Adamson T. E. Loudas N. B. Guo J. (2012). Functional association of Gdown1 with RNA polymerase II poised on human genes. *Mol. Cell.* 45 38–50. 10.1016/j.molcel.2011.10.022 22244331 PMC3259526

[B47] Cheng H. Capponi S. Wakeling E. Marchi E. Li Q. Zhao M. (2019). Missense variants in TAF1 and developmental phenotypes: challenges of determining pathogenicity. *Hum. Mutat.* Online ahead of print. 10.1002/humu.23936 PMC7187541 31646703

[B48] Chien R. Zeng W. Kawauchi S. Bender M. A. Santos R. Gregson H. C. (2011). Cohesin mediates chromatin interactions that regulate mammalian β-globin expression. *J. Biol. Chem.* 286 17870–17878. 10.1074/jbc.M110.207365 21454523 PMC3093862

[B49] Cho W. K. Spille J. H. Hecht M. Lee C. Li C. Grube V. (2018). Mediator and RNA polymerase II clusters associate in transcription-dependent condensates. *Science* 361 412–415. 10.1126/science.aar4199 29930094 PMC6543815

[B50] Chung H. L. Mao X. Wang H. Park Y. J. Marcogliese P. C. Rosenfeld J. A. (2020). De novo variants in CDK19 are associated with a syndrome involving intellectual disability and epileptic encephalopathy. *Am. J. Hum. Genet.* 106 717–725. 10.1016/j.ajhg.2020.04.001 32330417 PMC7212481

[B51] Cianfrocco M. A. Kassavetis G. A. Grob P. Fang J. Juven-Gershon T. Kadonaga J. T. (2013). Human TFIID binds to core promoter DNA in a reorganized structural state. *Cell* 152 120–131. 10.1016/j.cell.2012.12.005 23332750 PMC3552382

[B52] Coe B. P. Stessman H. A. F. Sulovari A. Geisheker M. R. Bakken T. E. Lake A. M. (2019). Neurodevelopmental disease genes implicated by de novo mutation and copy number variation morbidity. *Nat. Genet.* 51 106–116. 10.1038/s41588-018-0288-4 30559488 PMC6309590

[B53] Coffin G. S. Siris E. (1970). Mental retardation with absent fifth fingernail and terminal phalanx. *Am. J. Dis. Child* 119 433–439. 10.1001/archpedi.1970.02100050435009 5442442

[B54] Collins E. C. Appert A. Ariza-Mcnaughton L. Pannell R. Yamada Y. Rabbitts T. H. (2002). Mouse Af9 is a controller of embryo patterning, like Mll, whose human homologue fuses with Af9 after chromosomal translocation in leukemia. *Mol. Cell. Biol.* 22 7313–7324. 10.1128/MCB.22.20.7313-7324.2002 12242306 PMC139815

[B55] Daniels D. L. Ford M. Schwinn M. K. Benink H. Galbraith M. D. Amunugama R. (2013). Mutual exclusivity of MED12/MED12L, MED13/13L, and CDK8/19 paralogs revealed within the CDK-mediator kinase module. *J. Proteomics Bioinfom.* S2:004.

[B56] Darzacq X. Shav-Tal Y. De Turris V. Brody Y. Shenoy S. M. Phair R. D. (2007). *In vivo* dynamics of RNA polymerase II transcription. *Nat. Struct. Mol. Biol.* 14 796–806. 10.1038/nsmb1280 17676063 PMC4942130

[B57] Davidson I. F. Bauer B. Goetz D. Tang W. Wutz G. Peters J. M. (2019). DNA loop extrusion by human cohesin. *Science* 366 1338–1345. 10.1126/science.aaz3418 31753851

[B58] Dawson M. A. Prinjha R. K. Dittmann A. Giotopoulos G. Bantscheff M. Chan W. I. (2011). Inhibition of BET recruitment to chromatin as an effective treatment for MLL-fusion leukaemia. *Nature* 478 529–533. 21964340 10.1038/nature10509 PMC3679520

[B59] Day D. S. Zhang B. Stevens S. M. Ferrari F. Larschan E. N. Park P. J. (2016). Comprehensive analysis of promoter-proximal RNA polymerase II pausing across mammalian cell types. *Genome Biol.* 17:120. 10.1186/s13059-016-0984-2 27259512 PMC4893286

[B60] De Falco G. Bellan C. D’amuri A. Angeloni G. Leucci E. Giordano A. (2005). Cdk9 regulates neural differentiation and its expression correlates with the differentiation grade of neuroblastoma and PNET tumors. *Cancer Biol. Ther.* 4 277–281. 10.4161/cbt.4.3.1497 15753651

[B61] De Luca A. Esposito V. Baldi A. Claudio P. P. Fu Y. Caputi M. (1997). CDC2-related kinase PITALRE phosphorylates pRb exclusively on serine and is widely expressed in human tissues. *J. Cell. Physiol.* 172 265–273. 10.1002/(SICI)1097-4652(199708)172:2<265::AID-JCP13>3.0.CO;2-8 9258347

[B62] Deardorff M. A. Noon S. E. Krantz I. D. (1993). “Cornelia de lange syndrome,” in *GeneReviews(®)*, eds Adam M. P. Ardinger H. H. Pagon R. A. Wallace S. E. Bean L. J. H. Gripp K. W. (Seattle, WA: University of Washington).

[B63] Debacker K. Kooy R. F. (2007). Fragile sites and human disease. *Hum. Mol. Genet.* 16 Spec No. 2 R150–R158. 17567780 10.1093/hmg/ddm136

[B64] Deliu E. Arecco N. Morandell J. Dotter C. P. Contreras X. Girardot C. (2018). Haploinsufficiency of the intellectual disability gene SETD5 disturbs developmental gene expression and cognition. *Nat. Neurosci.* 21 1717–1727. 10.1038/s41593-018-0266-2 30455454

[B65] Deneault E. White S. H. Rodrigues D. C. Ross P. J. Faheem M. Zaslavsky K. (2018). Complete disruption of autism-susceptibility genes by gene editing predominantly reduces functional connectivity of isogenic human neurons. *Stem Cell Reports* 11 1211–1225. 10.1016/j.stemcr.2018.10.003 30392976 PMC6235011

[B66] Devaiah B. N. Lewis B. A. Cherman N. Hewitt M. C. Albrecht B. K. Robey P. G. (2012). BRD4 is an atypical kinase that phosphorylates serine2 of the RNA polymerase II carboxy-terminal domain. *Proc. Natl. Acad. Sci. U S A.* 109 6927–6932. 10.1073/pnas.1120422109 22509028 PMC3345009

[B67] Dey A. Chitsaz F. Abbasi A. Misteli T. Ozato K. (2003). The double bromodomain protein Brd4 binds to acetylated chromatin during interphase and mitosis. *Proc. Natl. Acad. Sci. U S A.* 100 8758–8763. 10.1073/pnas.1433065100 12840145 PMC166386

[B68] Ding N. Tomomori-Sato C. Sato S. Conaway R. C. Conaway J. W. Boyer T. G. (2009). MED19 and MED26 are synergistic functional targets of the RE1 silencing transcription factor in epigenetic silencing of neuronal gene expression. *J. Biol. Chem.* 284 2648–2656. 10.1074/jbc.M806514200 19049968 PMC2631966

[B69] Ding N. Zhou H. Esteve P. O. Chin H. G. Kim S. Xu X. (2008). Mediator links epigenetic silencing of neuronal gene expression with x-linked mental retardation. *Mol. Cell.* 31 347–359. 10.1016/j.molcel.2008.05.023 18691967 PMC2583939

[B70] Donner A. J. Ebmeier C. C. Taatjes D. J. Espinosa J. M. (2010). CDK8 is a positive regulator of transcriptional elongation within the serum response network. *Nat. Struct. Mol. Biol.* 17 194–201. 10.1038/nsmb.1752 20098423 PMC2920286

[B71] Donnio L. M. Bidon B. Hashimoto S. May M. Epanchintsev A. Ryan C. (2017). MED12-related XLID disorders are dose-dependent of immediate early genes (IEGs) expression. *Hum. Mol. Genet.* 26 2062–2075. 10.1093/hmg/ddx099 28369444

[B72] Durr K. Holzschuh J. Filippi A. Ettl A. K. Ryu S. Shepherd I. T. (2006). Differential roles of transcriptional mediator complex subunits Crsp34/Med27, Crsp150/Med14 and Trap100/Med24 during zebrafish retinal development. *Genetics* 174 693–705. 10.1534/genetics.105.055152 16582438 PMC1602071

[B73] Eash D. Waggoner D. Chung J. Stevenson D. Martin C. L. (2005). Calibration of 6q subtelomere deletions to define genotype/phenotype correlations. *Clin. Genet.* 67 396–403. 10.1111/j.1399-0004.2005.00424.x 15811006

[B74] Egloff S. (2021). CDK9 keeps RNA polymerase II on track. *Cell Mol. Life. Sci.* 78 5543–5567. 10.1007/s00018-021-03878-8 34146121 PMC8257543

[B75] Egloff S. Szczepaniak S. A. Dienstbier M. Taylor A. Knight S. Murphy S. (2010). The integrator complex recognizes a new double mark on the RNA polymerase II carboxyl-terminal domain. *J. Biol. Chem.* 285 20564–20569. 10.1074/jbc.M110.132530 20457598 PMC2898319

[B76] Egloff S. Van Herreweghe E. Kiss T. (2006). Regulation of polymerase II transcription by 7SK snRNA: two distinct RNA elements direct P-TEFb and HEXIM1 binding. *Mol. Cell. Biol.* 26 630–642. 10.1128/MCB.26.2.630-642.2006 16382153 PMC1346915

[B77] Elrod N. D. Henriques T. Huang K. L. Tatomer D. C. Wilusz J. E. Wagner E. J. (2019). The integrator complex attenuates promoter-proximal transcription at protein-coding genes. *Mol. Cell.* 76 738–752.e7. 10.1016/j.molcel.2019.10.034 31809743 PMC6952639

[B78] Euskirchen G. M. Auerbach R. K. Davidov E. Gianoulis T. A. Zhong G. Rozowsky J. (2011). Diverse roles and interactions of the SWI/SNF chromatin remodeling complex revealed using global approaches. *PLoS Genet* 7:e1002008. 10.1371/journal.pgen.1002008 PMC3048368 21408204

[B79] Fant C. B. Levandowski C. B. Gupta K. Maas Z. L. Moir J. Rubin J. D. (2020). TFIID enables RNA polymerase II promoter-proximal pausing. *Mol. Cell.* 78 785–793.e8. 10.1016/j.molcel.2020.03.008 32229306 PMC7245555

[B80] Fay A. Misulovin Z. Li J. Schaaf C. A. Gause M. Gilmour D. S. (2011). Cohesin selectively binds and regulates genes with paused RNA polymerase. *Curr. Biol.* 21 1624–1634. 10.1016/j.cub.2011.08.036 21962715 PMC3193539

[B81] Figueiredo T. Melo U. S. Pessoa A. L. Nobrega P. R. Kitajima J. P. Correa I. (2015). Homozygous missense mutation in MED25 segregates with syndromic intellectual disability in a large consanguineous family. *J. Med. Genet.* 52 123–127. 10.1136/jmedgenet-2014-102793 25527630

[B82] Filippakopoulos P. Qi J. Picaud S. Shen Y. Smith W. B. Fedorov O. (2010). Selective inhibition of BET bromodomains. *Nature* 468 1067–1073. 20871596 10.1038/nature09504 PMC3010259

[B83] Flynn G. A. Hirst M. C. Knight S. J. Macpherson J. N. Barber J. C. Flannery A. V. (1993). Identification of the FRAXE fragile site in two families ascertained for X linked mental retardation. *J. Med. Genet.* 30 97–100. 10.1136/jmg.30.2.97 8445629 PMC1016261

[B84] Francette A. M. Tripplehorn S. A. Arndt K. M. (2021). The Paf1 complex: a keystone of nuclear regulation operating at the interface of transcription and chromatin. *J. Mol. Biol.* 433:166979. 10.1016/j.jmb.2021.166979 33811920 PMC8184591

[B85] Frank K. M. Sharpless N. E. Gao Y. Sekiguchi J. M. Ferguson D. O. Zhu C. (2000). DNA ligase IV deficiency in mice leads to defective neurogenesis and embryonic lethality *via* the p53 pathway. *Mol. Cell.* 5 993–1002. 10.1016/s1097-2765(00)80264-6 10911993

[B86] Frey U. Frey S. Schollmeier F. Krug M. (1996). Influence of actinomycin D, a RNA synthesis inhibitor, on long-term potentiation in rat hippocampal neurons *in vivo* and in vitro. *J. Physiol.* 490(Pt 3), 703–711. 10.1113/jphysiol.1996.sp021179 8683469 PMC1158708

[B87] Frey U. Krug M. Brodemann R. Reymann K. Matthies H. (1989). Long-term potentiation induced in dendrites separated from rat’s CA1 pyramidal somata does not establish a late phase. *Neurosci. Lett.* 97 135–139. 10.1016/0304-3940(89)90152-3 2918996

[B88] Galbraith M. D. Allen M. A. Bensard C. L. Wang X. Schwinn M. K. Qin B. (2013). HIF1A employs CDK8-mediator to stimulate RNAPII elongation in response to hypoxia. *Cell* 153 1327–1339. 10.1016/j.cell.2013.04.048 23746844 PMC3681429

[B89] Gallant N. M. Baldwin E. Salamon N. Dipple K. M. Quintero-Rivera F. (2011). Pontocerebellar hypoplasia in association with de novo 19p13.11p13.12 microdeletion. *Am. J. Med. Genet. A* 155A 2871–2878. 10.1002/ajmg.a.34286 21994138

[B90] Gao R. Bao J. Yan H. Xie L. Qin W. Ning H. (2020). Competition between PAF1 and MLL1/COMPASS confers the opposing function of LEDGF/p75 in HIV latency and proviral reactivation. *Sci. Adv.* 6:eaaz8411. 10.1126/sciadv.aaz8411 32426500 PMC7220354

[B91] Gao Y. Ferguson D. O. Xie W. Manis J. P. Sekiguchi J. Frank K. M. (2000). Interplay of p53 and DNA-repair protein XRCC4 in tumorigenesis, genomic stability and development. *Nature* 404 897–900. 10.1038/35009138 10786799

[B92] Gardini A. Baillat D. Cesaroni M. Hu D. Marinis J. M. Wagner E. J. (2014). Integrator regulates transcriptional initiation and pause release following activation. *Mol. Cell.* 56 128–139. 10.1016/j.molcel.2014.08.004 25201415 PMC4292851

[B93] Gecz J. Gedeon A. K. Sutherland G. R. Mulley J. C. (1996). Identification of the gene FMR2, associated with FRAXE mental retardation. *Nat. Genet.* 13 105–108. 10.1038/ng0596-105 8673085

[B94] Gedeon A. K. Meinanen M. Ades L. C. Kaariainen H. Gecz J. Baker E. (1995). Overlapping submicroscopic deletions in Xq28 in two unrelated boys with developmental disorders: identification of a gene near FRAXE. *Am. J. Hum. Genet.* 56 907–914. 7536393 PMC1801213

[B95] Ghosh K. Tang M. Kumari N. Nandy A. Basu S. Mall D. P. (2018). Positive regulation of transcription by human ZMYND8 through its association with P-TEFb complex. *Cell Rep.* 24 2141–2154.e6. 10.1016/j.celrep.2018.07.064 30134174 PMC6152903

[B96] Gilchrist D. A. Dos Santos G. Fargo D. C. Xie B. Gao Y. Li L. (2010). Pausing of RNA polymerase II disrupts DNA-specified nucleosome organization to enable precise gene regulation. *Cell* 143 540–551. 10.1016/j.cell.2010.10.004 21074046 PMC2991113

[B97] Gilmour D. S. Lis J. T. (1986). RNA polymerase II interacts with the promoter region of the noninduced hsp70 gene in *Drosophila melanogaster* cells. *Mol. Cell. Biol.* 6 3984–3989. 10.1128/mcb.6.11.3984-3989.1986 3099167 PMC367162

[B98] Gokcezade J. Sienski G. Duchek P. (2014). Efficient CRISPR/Cas9 plasmids for rapid and versatile genome editing in *Drosophila*. *G3 (Bethesda)* 4 2279–2282. 10.1534/g3.114.014126 25236734 PMC4232553

[B99] Gressel S. Schwalb B. Decker T. M. Qin W. Leonhardt H. Eick D. (2017). CDK9-dependent RNA polymerase II pausing controls transcription initiation. *eLife* 6:e29736. 10.7554/eLife.29736 28994650 PMC5669633

[B100] Grozeva D. Carss K. Spasic-Boskovic O. Tejada M. I. Gecz J. Shaw M. (2015). Targeted next-generation sequencing analysis of 1,000 individuals with intellectual disability. *Hum. Mutat.* 36 1197–1204. 10.1002/humu.22901 26350204 PMC4833192

[B101] Gu Y. Nelson D. L. (2003). FMR2 function: insight from a mouse knockout model. *Cytogenet. Genome. Res.* 100 129–139. 10.1159/000072847 14526173

[B102] Gu Y. Mcilwain K. L. Weeber E. J. Yamagata T. Xu B. Antalffy B. A. (2002). Impaired conditioned fear and enhanced long-term potentiation in Fmr2 knock-out mice. *J. Neurosci.* 22 2753–2763. 10.1523/JNEUROSCI.22-07-02753.2002 11923441 PMC6758318

[B103] Gu Y. Shen Y. Gibbs R. A. Nelson D. L. (1996). Identification of FMR2, a novel gene associated with the FRAXE CCG repeat and CpG island. *Nat. Genet.* 13 109–113. 10.1038/ng0596-109 8673086

[B104] Gudmundsson S. Wilbe M. Filipek-Gorniok B. Molin A. M. Ekvall S. Johansson J. (2019). TAF1, associated with intellectual disability in humans, is essential for embryogenesis and regulates neurodevelopmental processes in zebrafish. *Sci. Rep.* 9:10730. 10.1038/s41598-019-46632-8 31341187 PMC6656882

[B105] Halevy A. Basel-Vanagaite L. Shuper A. Helman S. Har-Zahav A. Birk E. (2012). Microcephaly-thin corpus callosum syndrome maps to 8q23.2-q24.12. *Pediatr. Neurol.* 46 363–368. 10.1016/j.pediatrneurol.2012.03.014 22633631

[B106] Hamada N. Iwamoto I. Nishikawa M. Nagata K. I. (2021). Expression analyses of mediator complex subunit 13-Like: a responsible gene for neurodevelopmental disorders during mouse brain development. *Dev. Neurosci.* 43 43–52. 10.1159/000515188 33794529

[B107] Hashimoto S. Boissel S. Zarhrate M. Rio M. Munnich A. Egly J. M. (2011). MED23 mutation links intellectual disability to dysregulation of immediate early gene expression. *Science* 333 1161–1163. 10.1126/science.1206638 21868677

[B108] He N. Chan C. K. Sobhian B. Chou S. Xue Y. Liu M. (2011). Human polymerase-associated factor complex (PAFc) connects the super elongation complex (SEC) to RNA polymerase II on chromatin. *Proc. Natl. Acad. Sci. U S A.* 108 E636–E645. 10.1073/pnas.1107107108 21873227 PMC3169135

[B109] He N. Liu M. Hsu J. Xue Y. Chou S. Burlingame A. (2010). HIV-1 Tat and host AFF4 recruit two transcription elongation factors into a bifunctional complex for coordinated activation of HIV-1 transcription. *Mol. Cell.* 38 428–438. 10.1016/j.molcel.2010.04.013 20471948 PMC3085314

[B110] Hellman-Aharony S. Smirin-Yosef P. Halevy A. Pasmanik-Chor M. Yeheskel A. Har-Zahav A. (2013). Microcephaly thin corpus callosum intellectual disability syndrome caused by mutated TAF2. *Pediatr. Neurol.* 49 411–416.e1. 10.1016/j.pediatrneurol.2013.07.017 24084144

[B111] Hendrix D. A. Hong J. W. Zeitlinger J. Rokhsar D. S. Levine M. S. (2008). Promoter elements associated with RNA Pol II stalling in the *Drosophila embryo*. *Proc. Natl. Acad. Sci. U S A.* 105 7762–7767. 10.1073/pnas.0802406105 18505835 PMC2396556

[B112] Henriques T. Gilchrist D. A. Nechaev S. Bern M. Muse G. W. Burkholder A. (2013). Stable pausing by RNA polymerase II provides an opportunity to target and integrate regulatory signals. *Mol. Cell.* 52 517–528. 10.1016/j.molcel.2013.10.001 24184211 PMC3845087

[B113] Herrera F. J. Yamaguchi T. Roelink H. Tjian R. (2014). Core promoter factor TAF9B regulates neuronal gene expression. *eLife* 3:e02559. 10.7554/eLife.02559 25006164 PMC4083437

[B114] Herzfeld T. Nolte D. Grznarova M. Hofmann A. Schultze J. L. Muller U. (2013). X-linked dystonia parkinsonism syndrome (XDP, lubag): disease-specific sequence change DSC3 in TAF1/DYT3 affects genes in vesicular transport and dopamine metabolism. *Hum. Mol. Genet.* 22 941–951. 10.1093/hmg/dds499 23184149

[B115] Hiatt S. M. Lawlor J. M. J. Handley L. H. Ramaker R. C. Rogers B. B. Partridge E. C. (2021). Long-read genome sequencing for the molecular diagnosis of neurodevelopmental disorders. *HGG Adv.* 2:100023. 10.1016/j.xhgg.2021.100023 33937879 PMC8087252

[B116] Higashi T. L. Eickhoff P. Sousa J. S. Locke J. Nans A. Flynn H. R. (2020). A structure-based mechanism for DNA entry into the cohesin ring. *Mol. Cell.* 79 917–933.e9. 10.1016/j.molcel.2020.07.013 32755595 PMC7507959

[B117] Hillman M. A. Gecz J. (2001). Fragile XE-associated familial mental retardation protein 2 (FMR2) acts as a potent transcription activator. *J. Hum. Genet.* 46 251–259. 10.1007/s100380170074 11355014

[B118] Hiwatari M. Taki T. Taketani T. Taniwaki M. Sugita K. Okuya M. (2003). Fusion of an AF4-related gene, LAF4, to MLL in childhood acute lymphoblastic leukemia with t(2;11)(q11;q23). *Oncogene* 22 2851–2855. 10.1038/sj.onc.1206389 12743608

[B119] Honda S. Hayashi S. Kato M. Niida Y. Hayasaka K. Okuyama T. (2007). Clinical and molecular cytogenetic characterization of two patients with non-mutational aberrations of the FMR2 gene. *Am. J. Med. Genet. A* 143A 687–693. 10.1002/ajmg.a.31638 17343270

[B120] Houzelstein D. Bullock S. L. Lynch D. E. Grigorieva E. F. Wilson V. A. Beddington R. S. (2002). Growth and early postimplantation defects in mice deficient for the bromodomain-containing protein Brd4. *Mol. Cell. Biol.* 22 3794–3802. 10.1128/MCB.22.11.3794-3802.2002 11997514 PMC133820

[B121] Hu H. Haas S. A. Chelly J. Van Esch H. Raynaud M. De Brouwer A. P. (2016). X-exome sequencing of 405 unresolved families identifies seven novel intellectual disability genes. *Mol. Psychiatry* 21 133–148. 10.1038/mp.2014.193 25644381 PMC5414091

[B122] Hu S. Peng L. Xu C. Wang Z. Song A. Chen F. X. (2021). SPT5 stabilizes RNA polymerase II, orchestrates transcription cycles, and maintains the enhancer landscape. *Mol Cell.* 81 4425–4439.e6. 10.1016/j.molcel.2021.08.029 34534457

[B123] Hu X. Malik S. Negroiu C. C. Hubbard K. Velalar C. N. Hampton B. (2006). A Mediator-responsive form of metazoan RNA polymerase II. *Proc. Natl. Acad. Sci. U S A.* 103 9506–9511. 10.1073/pnas.0603702103 16769904 PMC1480437

[B124] Huang K.-L. Jee D. Stein C. B. Elrod N. D. Henriques T. Mascibroda L. G. (2020). Integrator recruits protein phosphatase 2A to prevent pause release and facilitate transcription termination. *Mol. Cell.* 80 345–358.e9. 10.1016/j.molcel.2020.08.016 32966759 PMC7660970

[B125] Huisman S. A. Redeker E. J. Maas S. M. Mannens M. M. Hennekam R. C. (2013). High rate of mosaicism in individuals with cornelia de lange syndrome. *J. Med. Genet.* 50 339–344. 10.1136/jmedgenet-2012-101477 23505322

[B126] Iossifov I. O’roak B. J. Sanders S. J. Ronemus M. Krumm N. Levy D. (2014). The contribution of de novo coding mutations to autism spectrum disorder. *Nature* 515 216–221. 10.1038/nature13908 25363768 PMC4313871

[B127] Itzen F. Greifenberg A. K. Bosken C. A. Geyer M. (2014). Brd4 activates P-TEFb for RNA polymerase II CTD phosphorylation. *Nucleic Acids Res.* 42 7577–7590. 10.1093/nar/gku449 24860166 PMC4081074

[B128] Izumi K. Nakato R. Zhang Z. Edmondson A. C. Noon S. Dulik M. C. (2015). Germline gain-of-function mutations in AFF4 cause a developmental syndrome functionally linking the super elongation complex and cohesin. *Nat. Genet.* 47 338–344. 10.1038/ng.3229 25730767 PMC4380798

[B129] Jaeger M. G. Schwalb B. Mackowiak S. D. Velychko T. Hanzl A. Imrichova H. (2020). Selective mediator dependence of cell-type-specifying transcription. *Nat. Genet.* 52 719–727. 10.1038/s41588-020-0635-0 32483291 PMC7610447

[B130] Jahani-Asl A. Cheng C. Zhang C. Bonni A. (2016). Pathogenesis of börjeson-forssman-lehmann syndrome: insights from PHF6 function. *Neurobiol. Dis.* 96 227–235. 10.1016/j.nbd.2016.09.011 27633282 PMC5102843

[B131] Jang M. K. Mochizuki K. Zhou M. Jeong H. S. Brady J. N. Ozato K. (2005). The bromodomain protein Brd4 is a positive regulatory component of P-TEFb and stimulates RNA polymerase II-dependent transcription. *Mol. Cell.* 19 523–534. 10.1016/j.molcel.2005.06.027 16109376

[B132] Jelsig A. M. Brasch-Andersen C. Kibaek M. Fagerberg C. R. (2012). A case of microdeletion of 19p13 with intellectual disability, hypertrichosis, synophrys, and protruding front teeth. *Eur. J. Med. Genet.* 55 564–567. 10.1016/j.ejmg.2012.06.009 22750323

[B133] Jensen D. R. Martin D. M. Gebarski S. Sahoo T. Brundage E. K. Chinault A. C. (2009). A novel chromosome 19p13.*12* deletion in a child with multiple congenital anomalies. *Am. J. Med. Genet. A* 149A 396–402. 10.1002/ajmg.a.32691 19215039 PMC2872113

[B134] Jeronimo C. Robert F. (2017). The mediator complex: at the nexus of RNA polymerase II transcription. *Trends Cell Biol.* 27 765–783. 10.1016/j.tcb.2017.07.001 28778422

[B135] Jiang Y. W. Veschambre P. Erdjument-Bromage H. Tempst P. Conaway J. W. Conaway R. C. (1998). Mammalian mediator of transcriptional regulation and its possible role as an end-point of signal transduction pathways. *Proc. Natl. Acad. Sci. U S A.* 95 8538–8543. 10.1073/pnas.95.15.8538 9671713 PMC21111

[B136] Jishage M. Malik S. Wagner U. Uberheide B. Ishihama Y. Hu X. (2012). Transcriptional regulation by Pol II(G) involving mediator and competitive interactions of Gdown1 and TFIIF with Pol II. *Mol. Cell.* 45 51–63. 10.1016/j.molcel.2011.12.014 22244332 PMC3259531

[B137] Jones S. Wang T.-L. Shih I.-M. Mao T.-L. Nakayama K. Roden R. (2010). Frequent mutations of chromatin remodeling gene ARID1A in ovarian clear cell carcinoma. *Science* 330 228–231. 10.1126/science.1196333 20826764 PMC3076894

[B138] Jonkers I. Kwak H. Lis J. T. (2014). Genome-wide dynamics of Pol II elongation and its interplay with promoter proximal pausing, chromatin, and exons. *eLife* 3:e02407. 10.7554/eLife.02407 24843027 PMC4001325

[B139] Judd J. Wojenski L. A. Wainman L. M. Tippens N. D. Rice E. J. Dziubek A. (2020). A rapid, sensitive, scalable method for Precision Run-On sequencing (PRO-seq). *bioRxiv [preprint]* 10.1101/2020.05.18.102277

[B140] Jung E. M. Moffat J. J. Liu J. Dravid S. M. Gurumurthy C. B. Kim W. Y. (2017). Arid1b haploinsufficiency disrupts cortical interneuron development and mouse behavior. *Nat. Neurosci.* 20 1694–1707. 10.1038/s41593-017-0013-0 29184203 PMC5726525

[B141] Jurynec M. J. Bai X. Bisgrove B. W. Jackson H. Nechiporuk A. Palu R. A. S. (2019). The Paf1 complex and P-TEFb have reciprocal and antagonist roles in maintaining multipotent neural crest progenitors. *Development* 146:dev180133. 10.1242/dev.180133 31784460 PMC6955205

[B142] Kagey M. H. Newman J. J. Bilodeau S. Zhan Y. Orlando D. A. Van Berkum N. L. (2010). Mediator and cohesin connect gene expression and chromatin architecture. *Nature* 467 430–435. 10.1038/nature09380 20720539 PMC2953795

[B143] Kahrizi K. Hu H. Hosseini M. Kalscheuer V. M. Fattahi Z. Beheshtian M. (2019). Effect of inbreeding on intellectual disability revisited by trio sequencing. *Clin. Genet.* 95 151–159. 10.1111/cge.13463 30315573

[B144] Kanno T. Kanno Y. Leroy G. Campos E. Sun H. W. Brooks S. R. (2014). BRD4 assists elongation of both coding and enhancer RNAs by interacting with acetylated histones. *Nat. Struct. Mol. Biol.* 21 1047–1057. 10.1038/nsmb.2912 25383670 PMC4720983

[B145] Kaufmann R. Straussberg R. Mandel H. Fattal-Valevski A. Ben-Zeev B. Naamati A. (2010). Infantile cerebral and cerebellar atrophy is associated with a mutation in the MED17 subunit of the transcription preinitiation mediator complex. *Am. J. Hum. Genet.* 87 667–670. 10.1016/j.ajhg.2010.09.016 20950787 PMC2978946

[B146] Kawauchi S. Calof A. L. Santos R. Lopez-Burks M. E. Young C. M. Hoang M. P. (2009). Multiple organ system defects and transcriptional dysregulation in the Nipbl+/- Mouse, a model of cornelia de lange syndrome. *PLoS Genetics* 5:e1000650. 10.1371/journal.pgen.1000650 PMC2730539 19763162

[B147] Kelso T. W. R. Porter D. K. Amaral M. L. Shokhirev M. N. Benner C. Hargreaves D. C. (2017). Chromatin accessibility underlies synthetic lethality of SWI/SNF subunits in ARID1A-mutant cancers. *eLife* 6:e30506. 10.7554/eLife.30506 28967863 PMC5643100

[B148] Kim J. Guermah M. Roeder R. G. (2010). The human PAF1 complex acts in chromatin transcription elongation both independently and cooperatively with SII/TFIIS. *Cell* 140 491–503. 10.1016/j.cell.2009.12.050 20178742 PMC2853908

[B149] Kim S. Y. Kim M. J. Kim S. J. Lee J. E. Chae J. H. Ko J. M. (2021). A case of CHOPS syndrome accompanied with moyamoya disease and systemic vasculopathy. *Brain Dev.* 43 454–458. 10.1016/j.braindev.2020.11.004 33248856

[B150] Kim Y. Shi Z. Zhang H. Finkelstein I. J. Yu H. (2019). Human cohesin compacts DNA by loop extrusion. *Science* 366 1345–1349. 10.1126/science.aaz4475 31780627 PMC7387118

[B151] Kline A. D. Grados M. Sponseller P. Levy H. P. Blagowidow N. Schoedel C. (2007). Natural history of aging in Cornelia de Lange syndrome. *Am. J. Med. Genet. C Semin. Med. Genet.* 145c 248–260. 10.1002/ajmg.c.30137 17640042 PMC4902018

[B152] Kline A. D. Moss J. F. Selicorni A. Bisgaard A.-M. Deardorff M. A. Gillett P. M. (2018). Diagnosis and management of Cornelia de Lange syndrome: first international consensus statement. *Nat. Rev. Genetics* 19 649–666. 29995837 10.1038/s41576-018-0031-0 PMC7136165

[B153] Knight S. J. Flannery A. V. Hirst M. C. Campbell L. Christodoulou Z. Phelps S. R. (1993). Trinucleotide repeat amplification and hypermethylation of a CpG island in FRAXE mental retardation. *Cell* 74 127–134. 10.1016/0092-8674(93)90300-f 8334699

[B154] Korb E. Herre M. Zucker-Scharff I. Darnell R. B. Allis C. D. (2015). BET protein Brd4 activates transcription in neurons and BET inhibitor Jq1 blocks memory in mice. *Nat. Neurosci.* 18 1464–1473. 10.1038/nn.4095 26301327 PMC4752120

[B155] Korb E. Herre M. Zucker-Scharff I. Gresack J. Allis C. D. Darnell R. B. (2017). Excess translation of epigenetic regulators contributes to fragile X syndrome and is alleviated by Brd4 Inhibition. *Cell* 170 1209–1223.e20. 10.1016/j.cell.2017.07.033 28823556 PMC5740873

[B156] Krall M. Htun S. Schnur R. E. Brooks A. S. Baker L. De Alba Campomanes A. (2019). Biallelic sequence variants in INTS1 in patients with developmental delays, cataracts, and craniofacial anomalies. *Eur. J. Hum. Genet.* 27 582–593. 10.1038/s41431-018-0298-9 30622326 PMC6460580

[B157] Krantz I. D. Mccallum J. Descipio C. Kaur M. Gillis L. A. Yaeger D. (2004). Cornelia de Lange syndrome is caused by mutations in NIPBL, the human homolog of *Drosophila melanogaster* Nipped-B. *Nat. Genet.* 36 631–635. 10.1038/ng1364 15146186 PMC4902017

[B158] Kuechler A. Zink A. M. Wieland T. Ludecke H. J. Cremer K. Salviati L. (2015). Loss-of-function variants of SETD5 cause intellectual disability and the core phenotype of microdeletion3p25.3 syndrome. *Eur. J. Hum. Genet.* 23 753–760. 10.1038/ejhg.2014.165 25138099 PMC4795044

[B159] Kwak H. Fuda N. F. Core L. J. Lis J. T. (2013). Precise maps of RNA polymerase reveal how promoters direct initiation and pausing. *Science* 339 950–953. 10.1126/science.1229386 23430654 PMC3974810

[B160] Lagha M. Bothma J. P. Esposito E. Ng S. Stefanik L. Tsui C. (2013). Paused Pol II coordinates tissue morphogenesis in the *Drosophila embryo*. *Cell* 153 976–987. 10.1016/j.cell.2013.04.045 23706736 PMC4257494

[B161] Lai F. Gardini A. Zhang A. Shiekhattar R. (2015). Integrator mediates the biogenesis of enhancer RNAs. *Nature* 525 399–403. 10.1038/nature14906 26308897 PMC4718573

[B162] Langer D. Martianov I. Alpern D. Rhinn M. Keime C. Dolle P. (2016). Essential role of the TFIID subunit TAF4 in murine embryogenesis and embryonic stem cell differentiation. *Nat. Commun.* 7:11063. 10.1038/ncomms11063 27026076 PMC4820908

[B163] Larochelle S. Amat R. Glover-Cutter K. Sansó M. Zhang C. Allen J. J. (2012). Cyclin-dependent kinase control of the initiation-to-elongation switch of RNA polymerase II. *Nat. Struct. Mol. Biol.* 19 1108–1115. 10.1038/nsmb.2399 23064645 PMC3746743

[B164] Latorre-Pellicer A. Gil-Salvador M. Parenti I. Lucia-Campos C. Trujillano L. Marcos-Alcalde I. (2021). Clinical relevance of postzygotic mosaicism in Cornelia de Lange syndrome and purifying selection of NIPBL variants in blood. *Sci. Rep.* 11:15459. 10.1038/s41598-021-94958-z 34326454 PMC8322329

[B165] Lawrence M. S. Stojanov P. Mermel C. H. Robinson J. T. Garraway L. A. Golub T. R. (2014). Discovery and saturation analysis of cancer genes across 21 tumour types. *Nature* 505 495–501. 10.1038/nature12912 24390350 PMC4048962

[B166] Leal A. Huehne K. Bauer F. Sticht H. Berger P. Suter U. (2009). Identification of the variant Ala335Val of MED25 as responsible for CMT2B2: molecular data, functional studies of the SH3 recognition motif and correlation between wild-type MED25 and PMP22 RNA levels in CMT1A animal models. *Neurogenetics* 10 275–287. 10.1007/s10048-009-0183-3 19290556 PMC2847151

[B167] Lee C. Li X. Hechmer A. Eisen M. Biggin M. D. Venters B. J. (2008). NELF and GAGA factor are linked to promoter-proximal pausing at many genes in *Drosophila*. *Mol. Cell. Biol.* 28 3290–3300. 10.1128/MCB.02224-07 18332113 PMC2423147

[B168] Lee Y. Choi I. Kim J. Kim K. (2016). DNA damage to human genetic disorders with neurodevelopmental defects. *J. Genet. Med.* 13 1–13. 10.5734/jgm.2016.13.1.1

[B169] Lee Y. Park D. Iyer V. R. (2017). The ATP-dependent chromatin remodeler Chd1 is recruited by transcription elongation factors and maintains H3K4me3/H3K36me3 domains at actively transcribed and spliced genes. *Nucleic Acids Res.* 45 7180–7190. 10.1093/nar/gkx321 28460001 PMC5499586

[B170] Lens Z. Cantrelle F. X. Peruzzini R. Hanoulle X. Dewitte F. Ferreira E. (2017). Solution structure of the n-terminal domain of mediator subunit MED26 and molecular characterization of its interaction with EAF1 and TAF7. *J. Mol. Biol.* 429 3043–3055. 10.1016/j.jmb.2017.09.001 28893534

[B171] Lesieur-Sebellin M. Capri Y. Grisval M. Courtin T. Burtz A. Thevenon J. (2021). Phenotype associated with TAF2 biallelic mutations: a clinical description of four individuals and review of the literature. *Eur. J. Med. Genet.* 64:104323. 10.1016/j.ejmg.2021.104323 34474177

[B172] Li D. Strong A. Shen K. M. Cassiman D. Van Dyck M. Linhares N. D. (2021a). De novo loss-of-function variants in X-linked MED12 are associated with Hardikar syndrome in females. *Genet. Med.* 23 637–644. 10.1038/s41436-020-01031-7 33244166

[B173] Li M. Qiu C. Bian Y. Shi D. Wang B. Ma Q. (2021b). SETD5 modulates homeostasis of hematopoietic stem cells by mediating RNA Polymerase II pausing in cooperation with HCF-1. *Leukemia* 36 1111–1122. 10.1038/s41375-021-01481-1 34853439 PMC8979820

[B174] Li-Kroeger D. Kanca O. Lee P. T. Cowan S. Lee M. T. Jaiswal M. (2018). An expanded toolkit for gene tagging based on MiMIC and scarless CRISPR tagging in Drosophila. *eLife* 7:e38709. 10.7554/eLife.38709 30091705 PMC6095692

[B175] Lin C. Garrett A. S. De Kumar B. Smith E. R. Gogol M. Seidel C. (2011). Dynamic transcriptional events in embryonic stem cells mediated by the super elongation complex (SEC). *Genes Dev.* 25 1486–1498. 10.1101/gad.2059211 21764852 PMC3143939

[B176] Lin C. Smith E. R. Takahashi H. Lai K. C. Martin-Brown S. Florens L. (2010). AFF4, a component of the ELL/P-TEFb elongation complex and a shared subunit of MLL chimeras, can link transcription elongation to leukemia. *Mol. Cell.* 37 429–437. 10.1016/j.molcel.2010.01.026 20159561 PMC2872029

[B177] Liu J. Baynam G. (2010). Cornelia de Lange syndrome. *Adv. Exp. Med. Biol.* 685 111–123. 20687500

[B178] Liu J. Krantz I. D. (2009). Cornelia de Lange syndrome, cohesin, and beyond. *Clin. Genet.* 76 303–314. 10.1111/j.1399-0004.2009.01271.x 19793304 PMC2853897

[B179] Liu K. Shen D. Shen J. Gao S. M. Li B. Wong C. (2017). The super elongation complex drives neural stem cell fate commitment. *Dev. Cell* 40 537–551.e6. 10.1016/j.devcel.2017.02.022 28350987

[B180] Liu X. Dai S. K. Liu P. P. Liu C. M. (2021). Arid1a regulates neural stem/progenitor cell proliferation and differentiation during cortical development. *Cell Prolif.* 54:e13124. 10.1111/cpr.13124 34562292 PMC8560606

[B181] Lower K. M. Turner G. Kerr B. A. Mathews K. D. Shaw M. A. Gedeon A. K. (2002). Mutations in PHF6 are associated with börjeson-forssman-lehmann syndrome. *Nat. Genet.* 32 661–665. 12415272 10.1038/ng1040

[B182] Lu X. Zhu X. Li Y. Liu M. Yu B. Wang Y. (2016). Multiple P-TEFbs cooperatively regulate the release of promoter-proximally paused RNA polymerase II. *Nucleic Acids Res.* 44 6853–6867. 10.1093/nar/gkw571 27353326 PMC5001612

[B183] Luna-Pelaez N. March-Diaz R. Ceballos-Chavez M. Guerrero-Martinez J. A. Grazioli P. Garcia-Gutierrez P. (2019). The cornelia de lange syndrome-associated factor NIPBL interacts with BRD4 ET domain for transcription control of a common set of genes. *Cell Death Dis.* 10:548. 10.1038/s41419-019-1792-x 31320616 PMC6639259

[B184] Luo Z. Lin C. Guest E. Garrett A. S. Mohaghegh N. Swanson S. (2012). The super elongation complex family of RNA polymerase II elongation factors: gene target specificity and transcriptional output. *Mol. Cell. Biol.* 32 2608–2617. 10.1128/MCB.00182-12 22547686 PMC3434493

[B185] Luo Z. Lin C. Woodfin A. R. Bartom E. T. Gao X. Smith E. R. (2016). Regulation of the imprinted Dlk1-Dio3 locus by allele-specific enhancer activity. *Genes Dev.* 30 92–101. 10.1101/gad.270413.115 26728555 PMC4701981

[B186] Ma C. Staudt L. M. (1996). LAF-4 encodes a lymphoid nuclear protein with transactivation potential that is homologous to AF-4, the gene fused to MLL in t(4;11) leukemias. *Blood* 87 734–745. 10.1182/blood.v87.2.734.bloodjournal872734 8555498

[B187] Maddirevula S. Alzahrani F. Al-Owain M. Al Muhaizea M. A. Kayyali H. R. Alhashem A. (2019). Autozygome and high throughput confirmation of disease genes candidacy. *Genet. Med.* 21 736–742. 10.1038/s41436-018-0138-x 30237576 PMC6752307

[B188] Makino S. Kaji R. Ando S. Tomizawa M. Yasuno K. Goto S. (2007). Reduced neuron-specific expression of the TAF1 gene is associated with X-linked dystonia-parkinsonism. *Am. J. Hum. Genet.* 80 393–406. 10.1086/512129 17273961 PMC1821114

[B189] Malovannaya A. Lanz R. B. Jung S. Y. Bulynko Y. Le N. T. Chan D. W. (2011). Analysis of the human endogenous coregulator complexome. *Cell* 145 787–799. 10.1016/j.cell.2011.05.006 21620140 PMC3131083

[B190] Mannini L. Cucco F. Quarantotti V. Krantz I. D. Musio A. (2013). Mutation spectrum and genotype-phenotype correlation in Cornelia de Lange syndrome. *Hum. Mutat.* 34 1589–1596. 10.1002/humu.22430 24038889 PMC3880228

[B191] Martianov I. Viville S. Davidson I. (2002). RNA polymerase II transcription in murine cells lacking the TATA binding protein. *Science* 298 1036–1039. 10.1126/science.1076327 12411709

[B192] Mashtalir N. D’avino A. R. Michel B. C. Luo J. Pan J. Otto J. E. (2018). Modular organization and assembly of SWI/SNF family chromatin remodeling complexes. *Cell* 175 1272–1288.e20. 10.1016/j.cell.2018.09.032 30343899 PMC6791824

[B193] Matrone G. Mullins J. J. Tucker C. S. Denvir M. A. (2016). Effects of cyclin dependent kinase 9 inhibition on zebrafish larvae. *Cell Cycle (Georgetown, Tex.)* 15 3060–3069. 10.1080/15384101.2016.1231283 27715402 PMC5134698

[B194] Meers M. P. Bryson T. D. Henikoff J. G. Henikoff S. (2019). Improved CUT&RUN chromatin profiling tools. *eLife* 8:e46314. 10.7554/eLife.46314 PMC6598765 31232687

[B195] Melko M. Nguyen L. S. Shaw M. Jolly L. Bardoni B. Gecz J. (2013). Loss of FMR2 further emphasizes the link between deregulation of immediate early response genes FOS and JUN and intellectual disability. *Hum. Mol. Genet.* 22 2984–2991. 10.1093/hmg/ddt155 23562910

[B196] Meng L. Isohanni P. Shao Y. Graham B. H. Hickey S. E. Brooks S. (2021). MED27 variants cause developmental delay, dystonia, and cerebellar hypoplasia. *Ann. Neurol.* 89 828–833. 10.1002/ana.26019 33443317

[B197] Messaoudi E. Ying S. W. Kanhema T. Croll S. D. Bramham C. R. (2002). Brain-derived neurotrophic factor triggers transcription-dependent, late phase long-term potentiation in vivo. *J. Neurosci.* 22 7453–7461. 10.1523/JNEUROSCI.22-17-07453.2002 12196567 PMC6757978

[B198] Metsu S. Rooms L. Rainger J. Taylor M. S. Bengani H. Wilson D. I. (2014). FRA2A is a CGG repeat expansion associated with silencing of AFF3. *PLoS Genet.* 10:e1004242. 10.1371/journal.pgen.1004242 PMC3998887 24763282

[B199] Moffat J. J. Jung E. M. Ka M. Jeon B. T. Lee H. Kim W. Y. (2021). Differential roles of ARID1B in excitatory and inhibitory neural progenitors in the developing cortex. *Sci. Rep.* 11:3856. 10.1038/s41598-021-82974-y 33594090 PMC7886865

[B200] Mondal K. Ramachandran D. Patel V. C. Hagen K. R. Bose P. Cutler D. J. (2012). Excess variants in AFF2 detected by massively parallel sequencing of males with autism spectrum disorder. *Hum. Mol. Genet.* 21 4356–4364. 10.1093/hmg/dds267 22773736 PMC3441129

[B201] Monies D. Abouelhoda M. Alsayed M. Alhassnan Z. Alotaibi M. Kayyali H. (2017). The landscape of genetic diseases in Saudi Arabia based on the first 1000 diagnostic panels and exomes. *Hum. Genet.* 136 921–939. 10.1007/s00439-017-1821-8 28600779 PMC5502059

[B202] Moore J. M. Oliver P. L. Finelli M. J. Lee S. Lickiss T. Molnar Z. (2014). Laf4/Aff3, a gene involved in intellectual disability, is required for cellular migration in the mouse cerebral cortex. *PLoS One* 9:e105933. 10.1371/journal.pone.0105933 PMC4146563 25162227

[B203] Moore S. J. Strain L. Cole G. F. Miedzybrodzka Z. Kelly K. F. Dean J. C. (1999). Fragile X syndrome with FMR1 and FMR2 deletion. *J. Med. Genet.* 36 565–566. 10424820 PMC1734406

[B204] Mueller C. L. Jaehning J. A. (2002). Ctr9, Rtf1, and Leo1 are components of the Paf1/RNA polymerase II complex. *Mol. Cell. Biol.* 22 1971–1980. 10.1128/MCB.22.7.1971-1980.2002 11884586 PMC133696

[B205] Mueller D. Garcia-Cuellar M. P. Bach C. Buhl S. Maethner E. Slany R. K. (2009). Misguided transcriptional elongation causes mixed lineage leukemia. *PLoS Biol.* 7:e1000249. 10.1371/journal.pbio.1000249 PMC2774266 19956800

[B206] Muhar M. Ebert A. Neumann T. Umkehrer C. Jude J. Wieshofer C. (2018). SLAM-seq defines direct gene-regulatory functions of the BRD4-MYC axis. *Science* 360:800. 10.1126/science.aao2793 29622725 PMC6409205

[B207] Mukhopadhyay A. Kramer J. M. Merkx G. Lugtenberg D. Smeets D. F. Oortveld M. A. (2010). CDK19 is disrupted in a female patient with bilateral congenital retinal folds, microcephaly and mild mental retardation. *Hum. Genet.* 128 281–291. 10.1007/s00439-010-0848-x 20563892 PMC2921488

[B208] Munoz-Fuentes V. Cacheiro P. Meehan T. F. Aguilar-Pimentel J. A. Brown S. D. M. Flenniken A. M. (2018). The International Mouse Phenotyping Consortium (IMPC): a functional catalogue of the mammalian genome that informs conservation. *Conserv. Genet.* 19 995–1005. 10.1007/s10592-018-1072-9 30100824 PMC6061128

[B209] Murthy D. S. Teebi A. S. Sundareshan T. S. Al-Awadi S. A. (1990). Familial fragile secondary constriction on chromosome 2 (2q11) with unusual features and psychomotor retardation. *Indian J. Pediatr.* 57 257–260. 10.1007/BF02722098 2246023

[B210] Najmabadi H. Hu H. Garshasbi M. Zemojtel T. Abedini S. S. Chen W. (2011). Deep sequencing reveals 50 novel genes for recessive cognitive disorders. *Nature* 478 57–63. 10.1038/nature10423 21937992

[B211] Nakagawa T. Hattori S. Nobuta R. Kimura R. Nakagawa M. Matsumoto M. (2020). The autism-related protein SETD5 controls neural cell proliferation through epigenetic regulation of rDNA expression. *iScience* 23:101030. 10.1016/j.isci.2020.101030 32299058 PMC7160574

[B212] Newkirk D. A. Chen Y. Y. Chien R. Zeng W. Biesinger J. Flowers E. (2017). The effect of Nipped-B-like (Nipbl) haploinsufficiency on genome-wide cohesin binding and target gene expression: modeling Cornelia de Lange syndrome. *Clin. Epigenet.* 9:89. 10.1186/s13148-017-0391-x 28855971 PMC5574093

[B213] Nguyen P. V. Abel T. Kandel E. R. (1994). Requirement of a critical period of transcription for induction of a late phase of LTP. *Science* 265 1104–1107. 10.1126/science.8066450 8066450

[B214] Niranjan T. S. Skinner C. May M. Turner T. Rose R. Stevenson R. (2015). Affected kindred analysis of human X chromosome exomes to identify novel X-linked intellectual disability genes. *PLoS One* 10:e0116454. 10.1371/journal.pone.0116454 PMC4332666 25679214

[B215] Nishina S. Hosono K. Ishitani S. Kosaki K. Yokoi T. Yoshida T. (2021). Biallelic CDK9 variants as a cause of a new multiple-malformation syndrome with retinal dystrophy mimicking the CHARGE syndrome. *J. Hum. Genet.* 66 1021–1027. 10.1038/s10038-021-00909-x 33640901 PMC8472910

[B216] Nizon M. Laugel V. Flanigan K. M. Pastore M. Waldrop M. A. Rosenfeld J. A. (2019). Variants in MED12L, encoding a subunit of the mediator kinase module, are responsible for intellectual disability associated with transcriptional defect. *Genet. Med.* 21 2713–2722. 10.1038/s41436-019-0557-3 31155615 PMC7243155

[B217] Nogales E. Patel A. B. Louder R. K. (2017). Towards a mechanistic understanding of core promoter recognition from cryo-EM studies of human TFIID. *Curr. Opin. Struct. Biol.* 47 60–66. 10.1016/j.sbi.2017.05.015 28624568 PMC5723225

[B218] Nojima T. Gomes T. Grosso A. R. F. Kimura H. Dye M. J. (2015). Mammalian NET-Seq reveals genome-wide nascent transcription coupled to RNA processing. *Cell* 161 526–540. 10.1016/j.cell.2015.03.027 25910207 PMC4410947

[B219] Oegema R. Baillat D. Schot R. Van Unen L. M. Brooks A. Kia S. K. (2017). Human mutations in integrator complex subunits link transcriptome integrity to brain development. *PLoS Genet.* 13:e1006809. 10.1371/journal.pgen.1006809 PMC5466333 28542170

[B220] Oh H. R. An C. H. Yoo N. J. Lee S. H. (2017). Frameshift mutations in the mononucleotide repeats of TAF1 and TAF1L genes in gastric and colorectal cancers with regional heterogeneity. *Pathol. Oncol. Res.* 23 125–130. 10.1007/s12253-016-0107-0 27571988

[B221] Oka Y. Suzuki K. Yamauchi M. Mitsutake N. Yamashita S. (2011). Recruitment of the cohesin loading factor NIPBL to DNA double-strand breaks depends on MDC1. RNF168 and HP1γ in human cells. *Biochem. Biophys. Res. Commun.* 411 762–767. 10.1016/j.bbrc.2011.07.021 21784059

[B222] Okamoto N. Arai H. Onishi T. Mizuguchi T. Matsumoto N. (2020). Intellectual disability and dysmorphic features in male siblings arising from a novel TAF1 mutation. *Congenit Anom (Kyoto)* 60 40–41. 10.1111/cga.12330 30805980

[B223] Oliver P. L. Bitoun E. Clark J. Jones E. L. Davies K. E. (2004). Mediation of Af4 protein function in the cerebellum by Siah proteins. *Proc. Natl. Acad. Sci. U S A.* 101 14901–14906. 10.1073/pnas.0406196101 15459319 PMC522018

[B224] Olley G. Ansari M. Bengani H. Grimes G. R. Rhodes J. Von Kriegsheim A. (2018). BRD4 interacts with NIPBL and BRD4 is mutated in a Cornelia de Lange-like syndrome. *Nat. Genet.* 50 329–332. 10.1038/s41588-018-0042-y 29379197 PMC6469577

[B225] O’Rawe J. A. Wu Y. Dorfel M. J. Rope A. F. Au P. Y. Parboosingh J. S. (2015). TAF1 variants are associated with dysmorphic features, intellectual disability, and neurological manifestations. *Am. J. Hum. Genet.* 97 922–932. 10.1016/j.ajhg.2015.11.005 26637982 PMC4678794

[B226] Osipovich A. B. Gangula R. Vianna P. G. Magnuson M. A. (2016). Setd5 is essential for mammalian development and the co-transcriptional regulation of histone acetylation. *Development* 143 4595–4607. 10.1242/dev.141465 27864380 PMC5201031

[B227] Parenti I. Teresa-Rodrigo M. E. Pozojevic J. Ruiz Gil S. Bader I. Braunholz D. (2017). Mutations in chromatin regulators functionally link Cornelia de Lange syndrome and clinically overlapping phenotypes. *Hum. Genet.* 136 307–320. 10.1007/s00439-017-1758-y 28120103

[B228] Perez-Cadahia B. Drobic B. Davie J. R. (2011). Activation and function of immediate-early genes in the nervous system. *Biochem. Cell Biol.* 89 61–73. 10.1139/O10-138 21326363

[B229] Pilarowski G. O. Vernon H. J. Applegate C. D. Boukas L. Cho M. T. Gurnett C. A. (2018). Missense variants in the chromatin remodeler CHD1 are associated with neurodevelopmental disability. *J. Med. Genet.* 55 561–566. 10.1136/jmedgenet-2017-104759 28866611 PMC5834353

[B230] Plassche S. V. Brouwer A. P. (2021). MED12-Related (Neuro)developmental disorders: a question of causality. *Genes (Basel)* 12:663. 10.3390/genes12050663 33925166 PMC8146938

[B231] Polla D. L. Bhoj E. J. Verheij J. Wassink-Ruiter J. S. K. Reis A. Deshpande C. (2021). De novo variants in MED12 cause X-linked syndromic neurodevelopmental disorders in 18 females. *Genet. Med.* 23 645–652. 10.1038/s41436-020-01040-6 33244165

[B232] Poot M. (2020). Mutations in mediator complex genes CDK8, MED12, MED13, and MEDL13 mediate overlapping developmental syndromes. *Mol. Syndromol.* 10 239–242. 10.1159/000502346 32021594 PMC6997798

[B233] Powis Z. Farwell Hagman K. D. Mroske C. Mcwalter K. Cohen J. S. Colombo R. (2018). Expansion and further delineation of the SETD5 phenotype leading to global developmental delay, variable dysmorphic features, and reduced penetrance. *Clin. Genet.* 93 752–761. 10.1111/cge.13132 28881385

[B234] Pramparo T. Grosso S. Messa J. Zatterale A. Bonaglia M. C. Chessa L. (2005). Loss-of-function mutation of the AF9/MLLT3 gene in a girl with neuromotor development delay, cerebellar ataxia, and epilepsy. *Hum. Genet.* 118 76–81. 10.1007/s00439-005-0004-1 16001262

[B235] Qiao Y. Wang X. Wang R. Li Y. Yu F. Yang X. (2015). AF9 promotes hESC neural differentiation through recruiting TET2 to neurodevelopmental gene loci for methylcytosine hydroxylation. *Cell Discov.* 1:15017. 10.1038/celldisc.2015.17 27462416 PMC4860857

[B236] Raab J. R. Resnick S. Magnuson T. (2015). Genome-Wide transcriptional regulation mediated by biochemically distinct SWI/SNF complexes. *PLoS Genet* 11:e1005748. 10.1371/journal.pgen.1005748 PMC4699898 26716708

[B237] Rahman S. Sowa M. E. Ottinger M. Smith J. A. Shi Y. Harper J. W. (2011). The Brd4 extraterminal domain confers transcription activation independent of pTEFb by recruiting multiple proteins, including NSD3. *Mol. Cell. Biol.* 31 2641–2652. 10.1128/MCB.01341-10 21555454 PMC3133372

[B238] Raible S. E. Mehta D. Bettale C. Fiordaliso S. Kaur M. Medne L. (2019). Clinical and molecular spectrum of CHOPS syndrome. *Am. J. Med. Genet. A* 179 1126–1138. 10.1002/ajmg.a.61174 31058441 PMC7473581

[B239] Remeseiro S. Cuadrado A. Kawauchi S. Calof A. L. Lander A. D. Losada A. (2013). Reduction of Nipbl impairs cohesin loading locally and affects transcription but not cohesion-dependent functions in a mouse model of Cornelia de Lange Syndrome. *Biochim. Biophys. Acta* 1832 2097–2102. 10.1016/j.bbadis.2013.07.020 23920377 PMC3825806

[B240] Rengachari S. Schilbach S. Aibara S. Dienemann C. Cramer P. (2021). Structure of the human Mediator–RNA polymerase II pre-initiation complex. *Nature* 594 129–133. 10.1038/s41586-021-03555-7 33902108

[B241] Rentas S. Rathi K. S. Kaur M. Raman P. Krantz I. D. Sarmady M. (2020). Diagnosing Cornelia de Lange syndrome and related neurodevelopmental disorders using RNA sequencing. *Genet. Med.* 22 927–936. 10.1038/s41436-019-0741-5 31911672

[B242] Risheg H. Graham J. M. Jr. Clark R. D. Rogers R. C. Opitz J. M. (2007). A recurrent mutation in MED12 leading to R961W causes Opitz-Kaveggia syndrome. *Nat. Genet.* 39 451–453. 10.1038/ng1992 17334363

[B243] Rk C. Y. Merico D. Bookman M. Howe L. J. Thiruvahindrapuram B. Patel R. V. (2017). Whole genome sequencing resource identifies 18 new candidate genes for autism spectrum disorder. *Nat. Neurosci.* 20 602–611. 10.1038/nn.4524 28263302 PMC5501701

[B244] Robinson P. J. Trnka M. J. Bushnell D. A. Davis R. E. Mattei P. J. Burlingame A. L. (2016). Structure of a complete mediator-RNA polymerase II pre-initiation complex. *Cell* 166 1411–1422.e16. 10.1016/j.cell.2016.08.050 27610567 PMC5589196

[B245] Roeder R. G. (1996). The role of general initiation factors in transcription by RNA polymerase II. *Trends Biochem. Sci.* 21 327–335. 10.1016/s0968-0004(96)10050-5 8870495

[B246] Rooms L. Reyniers E. Scheers S. Van Luijk R. Wauters J. Van Aerschot L. (2006). TBP as a candidate gene for mental retardation in patients with subtelomeric 6q deletions. *Eur. J. Hum. Genet.* 14 1090–1096. 10.1038/sj.ejhg.5201674 16773126

[B247] Rougvie A. E. Lis J. T. (1988). The RNA polymerase II molecule at the 5’ end of the uninduced hsp70 gene of D. melanogaster is transcriptionally engaged. *Cell* 54 795–804. 10.1016/s0092-8674(88)91087-2 3136931

[B248] Rubtsova M. P. Vasilkova D. P. Moshareva M. A. Malyavko A. N. Meerson M. B. Zatsepin T. S. (2019). Integrator is a key component of human telomerase RNA biogenesis. *Sci. Rep.* 9:1701. 10.1038/s41598-018-38297-6 30737432 PMC6368637

[B249] Sabari B. R. Dall’agnese A. Boija A. Klein I. A. Coffey E. L. Shrinivas K. (2018). Coactivator condensation at super-enhancers links phase separation and gene control. *Science* 361:eaar3958. 10.1126/science.aar3958 29930091 PMC6092193

[B250] Saha R. N. Wissink E. M. Bailey E. R. Zhao M. Fargo D. C. Hwang J.-Y. (2011). Rapid activity-induced transcription of Arc and other IEGs relies on poised RNA polymerase II. *Nat. Neurosci.* 14 848–856. 10.1038/nn.2839 21623364 PMC3125443

[B251] Sahoo T. Theisen A. Marble M. Tervo R. Rosenfeld J. A. Torchia B. S. (2011). Microdeletion of Xq28 involving the AFF2 (FMR2) gene in two unrelated males with developmental delay. *Am. J. Med. Genet. A* 155A 3110–3115. 10.1002/ajmg.a.34345 22065534

[B252] Santen G. W. Aten E. Sun Y. Almomani R. Gilissen C. Nielsen M. (2012). Mutations in SWI/SNF chromatin remodeling complex gene ARID1B cause Coffin-Siris syndrome. *Nat. Genet.* 44 379–380. 10.1038/ng.2217 22426309

[B253] Santen G. W. Aten E. Vulto-Van Silfhout A. T. Pottinger C. Van Bon B. W. Van Minderhout I. J. (2013). Coffin-Siris syndrome and the BAF complex: genotype-phenotype study in 63 patients. *Hum. Mutat.* 34 1519–1528. 10.1002/humu.22394 23929686

[B254] Schaaf C. A. Kwak H. Koenig A. Misulovin Z. Gohara D. W. Watson A. (2013). Genome-wide control of RNA polymerase II activity by cohesin. *PLoS Genet* 9:e1003382. 10.1371/journal.pgen.1003382 PMC3605059 23555293

[B255] Schiano C. Casamassimi A. Rienzo M. De Nigris F. Sommese L. Napoli C. (2014). Involvement of Mediator complex in malignancy. *Biochim. Biophys. Acta* 1845 66–83. 10.1016/j.bbcan.2013.12.001 24342527

[B256] Schroder S. Cho S. Zeng L. Zhang Q. Kaehlcke K. Mak L. (2012). Two-pronged binding with bromodomain-containing protein 4 liberates positive transcription elongation factor b from inactive ribonucleoprotein complexes. *J. Biol. Chem.* 287 1090–1099. 10.1074/jbc.M111.282855 22084242 PMC3256921

[B257] Schwartz C. E. Tarpey P. S. Lubs H. A. Verloes A. May M. M. Risheg H. (2007). The original Lujan syndrome family has a novel missense mutation (p.N1007S) in the MED12 gene. *J. Med. Genet.* 44 472–477. 10.1136/jmg.2006.048637 17369503 PMC2597996

[B258] Sessa A. Fagnocchi L. Mastrototaro G. Massimino L. Zaghi M. Indrigo M. (2019). SETD5 regulates chromatin methylation state and preserves global transcriptional fidelity during brain development and neuronal wiring. *Neuron* 104 271–289.e13. 10.1016/j.neuron.2019.07.013 31515109

[B259] Shaheen R. Patel N. Shamseldin H. Alzahrani F. Al-Yamany R. ALMoisheer A. (2016). Accelerating matchmaking of novel dysmorphology syndromes through clinical and genomic characterization of a large cohort. *Genet. Med.* 18 686–695. 10.1038/gim.2015.147 26633546

[B260] Shao W. Alcantara S. G. Zeitlinger J. (2019). Reporter-ChIP-nexus reveals strong contribution of the *Drosophila* initiator sequence to RNA polymerase pausing. *eLife* 8:e41461. 10.7554/eLife.41461 31021316 PMC6483594

[B261] Shao W. Zeitlinger J. (2017). Paused RNA polymerase II inhibits new transcriptional initiation. *Nat. Genet.* 49 1045–1051. 10.1038/ng.3867 28504701

[B262] Shi J. Vakoc C. R. (2014). The mechanisms behind the therapeutic activity of BET bromodomain inhibition. *Mol. Cell.* 54 728–736. 10.1016/j.molcel.2014.05.016 24905006 PMC4236231

[B263] Shi X. Chang M. Wolf A. J. Chang C. H. Frazer-Abel A. A. Wade P. A. (1997). Cdc73p and Paf1p are found in a novel RNA polymerase II-containing complex distinct from the Srbp-containing holoenzyme. *Mol. Cell. Biol.* 17 1160–1169. 10.1128/MCB.17.3.1160 9032243 PMC231841

[B264] Shi Z. Gao H. Bai X. C. Yu H. (2020). Cryo-EM structure of the human cohesin-NIPBL-DNA complex. *Science* 368 1454–1459. 10.1126/science.abb0981 32409525

[B265] Shimizu D. Sakamoto R. Yamoto K. Saitsu H. Fukami M. Nishimura G. (2019). De novo AFF3 variant in a patient with mesomelic dysplasia with foot malformation. *J. Hum. Genet.* 64 1041–1044. 10.1038/s10038-019-0650-0 31388108

[B266] Skaar J. R. Ferris A. L. Wu X. Saraf A. Khanna K. K. Florens L. (2015). The Integrator complex controls the termination of transcription at diverse classes of gene targets. *Cell Res.* 25 288–305. 10.1038/cr.2015.19 25675981 PMC4349240

[B267] Skene P. J. Hernandez A. E. Groudine M. Henikoff S. (2014). The nucleosomal barrier to promoter escape by RNA polymerase II is overcome by the chromatin remodeler Chd1. *eLife* 3:e02042. 10.7554/eLife.02042 24737864 PMC3983905

[B268] Smith E. Lin C. Shilatifard A. (2011). The super elongation complex (SEC) and MLL in development and disease. *Genes Dev.* 25 661–672. 10.1101/gad.2015411 21460034 PMC3070929

[B269] Smol T. Petit F. Piton A. Keren B. Sanlaville D. Afenjar A. (2018). MED13L-related intellectual disability: involvement of missense variants and delineation of the phenotype. *Neurogenetics* 19 93–103. 10.1007/s10048-018-0541-0 29511999

[B270] Snijders Blok L. Hiatt S. M. Bowling K. M. Prokop J. W. Engel K. L. Cochran J. N. (2018). De novo mutations in MED13, a component of the Mediator complex, are associated with a novel neurodevelopmental disorder. *Hum. Genet.* 137 375–388. 10.1007/s00439-018-1887-y 29740699 PMC5973976

[B271] Soutourina J. (2018). Transcription regulation by the Mediator complex. *Nat. Rev. Mol. Cell Biol.* 19 262–274. 10.1038/nrm.2017.115 29209056

[B272] Soutourina J. Werner M. (2014). A novel link of mediator with DNA repair. *Cell Cycle* 13 1362–1363. 10.4161/cc.28749 24698781 PMC4050128

[B273] Srivastava S. Kulshreshtha R. (2021). Insights into the regulatory role and clinical relevance of mediator subunit, MED12, in human diseases. *J. Cell. Physiol.* 236 3163–3177. 10.1002/jcp.30099 33174211

[B274] Stadelmayer B. Micas G. Gamot A. Martin P. Malirat N. Koval S. (2014). Integrator complex regulates NELF-mediated RNA polymerase II pause/release and processivity at coding genes. *Nat. Commun.* 5:5531. 10.1038/ncomms6531 25410209 PMC4263189

[B275] Stahl E. A. Raychaudhuri S. Remmers E. F. Xie G. Eyre S. Thomson B. P. (2010). Genome-wide association study meta-analysis identifies seven new rheumatoid arthritis risk loci. *Nat. Genet.* 42 508–514. 10.1038/ng.582 20453842 PMC4243840

[B276] Steichen-Gersdorf E. Gassner I. Superti-Furga A. Ullmann R. Stricker S. Klopocki E. (2008). Triangular tibia with fibular aplasia associated with a microdeletion on 2q11.2 encompassing LAF4. *Clin. Genet.* 74 560–565. 10.1111/j.1399-0004.2008.01050.x 18616733

[B277] Stenson P. D. Mort M. Ball E. V. Shaw K. Phillips A. Cooper D. N. (2014). The human gene mutation database: building a comprehensive mutation repository for clinical and molecular genetics, diagnostic testing and personalized genomic medicine. *Hum. Genet.* 133 1–9. 10.1007/s00439-013-1358-4 24077912 PMC3898141

[B278] Stettner G. M. Shoukier M. Hoger C. Brockmann K. Auber B. (2011). Familial intellectual disability and autistic behavior caused by a small FMR2 gene deletion. *Am. J. Med. Genet. A* 155A 2003–2007. 10.1002/ajmg.a.34122 21739600

[B279] Steurer B. Janssens R. C. Geverts B. Geijer M. E. Wienholz F. Theil A. F. (2018). Live-cell analysis of endogenous GFP-RPB1 uncovers rapid turnover of initiating and promoter-paused RNA Polymerase II. *Proc. Natl. Acad. Sci. U S A.* 115 E4368–E4376. 10.1073/pnas.1717920115 29632207 PMC5948963

[B280] Striano P. Elia M. Castiglia L. Galesi O. Pelligra S. Striano S. (2005). A t(4;9)(q34;p22) translocation associated with partial epilepsy, mental retardation, and dysmorphism. *Epilepsia* 46 1322–1324. 10.1111/j.1528-1167.2005.64304.x 16060948

[B281] Strissel P. L. Strick R. Tomek R. J. Roe B. A. Rowley J. D. Zeleznik-Le N. J. (2000). DNA structural properties of AF9 are similar to MLL and could act as recombination hot spots resulting in MLL/AF9 translocations and leukemogenesis. *Hum. Mol. Genet.* 9 1671–1679. 10.1093/hmg/9.11.1671 10861294

[B282] Takahashi H. Parmely T. J. Sato S. Tomomori-Sato C. Banks C. A. Kong S. E. (2011). Human mediator subunit MED26 functions as a docking site for transcription elongation factors. *Cell* 146 92–104. 10.1016/j.cell.2011.06.005 21729782 PMC3145325

[B283] Tatomer D. C. Elrod N. D. Liang D. Xiao M. S. Jiang J. Z. Jonathan M. (2019). The Integrator complex cleaves nascent mRNAs to attenuate transcription. *Genes Dev.* 33 1525–1538. 10.1101/gad.330167.119 31530651 PMC6824465

[B284] Tawamie H. Martianov I. Wohlfahrt N. Buchert R. Mengus G. Uebe S. (2017). Hypomorphic pathogenic variants in TAF13 are associated with autosomal-recessive intellectual disability and microcephaly. *Am. J. Hum. Genet.* 100 555–561. 10.1016/j.ajhg.2017.01.032 28257693 PMC5339287

[B285] Thevenon J. Duffourd Y. Masurel-Paulet A. Lefebvre M. Feillet F. El Chehadeh-Djebbar S. (2016). Diagnostic odyssey in severe neurodevelopmental disorders: toward clinical whole-exome sequencing as a first-line diagnostic test. *Clin. Genet.* 89 700–707. 10.1111/cge.12732 26757139

[B286] Thirman M. J. Levitan D. A. Kobayashi H. Simon M. C. Rowley J. D. (1994). Cloning of ELL, a gene that fuses to MLL in a t(11;19)(q23;p13.1) in acute myeloid leukemia. *Proc. Natl. Acad. Sci. U S A.* 91 12110–12114. 10.1073/pnas.91.25.12110 7991593 PMC45386

[B287] Timms K. M. Bondeson M. L. Ansari-Lari M. A. Lagerstedt K. Muzny D. M. Dugan-Rocha S. P. (1997). Molecular and phenotypic variation in patients with severe Hunter syndrome. *Hum. Mol. Genet.* 6 479–486. 10.1093/hmg/6.3.479 9147653

[B288] To M. D. Faseruk S. A. Gokgoz N. Pinnaduwage D. Done S. J. Andrulis I. L. (2005). LAF-4 is aberrantly expressed in human breast cancer. *Int. J. Cancer* 115 568–574. 10.1002/ijc.20881 15704140

[B289] Tonkin E. T. Wang T. J. Lisgo S. Bamshad M. J. Strachan T. (2004). NIPBL, encoding a homolog of fungal Scc2-type sister chromatid cohesion proteins and fly Nipped-B, is mutated in Cornelia de Lange syndrome. *Nat. Genet.* 36 636–641. 10.1038/ng1363 15146185

[B290] Torring P. M. Larsen M. J. Brasch-Andersen C. Krogh L. N. Kibaek M. Laulund L. (2019). Is MED13L-related intellectual disability a recognizable syndrome? *Eur. J. Med. Genet.* 62 129–136. 10.1016/j.ejmg.2018.06.014 29959045

[B291] Trehan A. Brady J. M. Maduro V. Bone W. P. Huang Y. Golas G. A. (2015). MED23-associated intellectual disability in a non-consanguineous family. *Am. J. Med. Genet. A* 167 1374–1380. 10.1002/ajmg.a.37047 25845469 PMC5671761

[B292] Trizzino M. Barbieri E. Petracovici A. Wu S. Welsh S. A. Owens T. A. (2018). The tumor suppressor ARID1A controls global transcription *via* pausing of RNA polymerase II. *Cell Rep.* 23 3933–3945. 10.1016/j.celrep.2018.05.097 29949775 PMC6146183

[B293] Tsai K. L. Tomomori-Sato C. Sato S. Conaway R. C. Conaway J. W. Asturias F. J. (2014). Subunit architecture and functional modular rearrangements of the transcriptional mediator complex. *Cell* 157 1430–1444. 10.1016/j.cell.2014.05.015 24882805 PMC4104964

[B294] Tsai K. L. Yu X. Gopalan S. Chao T. C. Zhang Y. Florens L. (2017). Mediator structure and rearrangements required for holoenzyme formation. *Nature* 544 196–201. 10.1038/nature21393 28241144 PMC6692119

[B295] Tsujino K. Okuzaki Y. Hibino N. Kawamura K. Saito S. Ozaki Y. (2019). Identification of transgene integration site and anatomical properties of fluorescence intensity in a EGFP transgenic chicken line. *Dev. Growth. Differ.* 61 393–401. 10.1111/dgd.12631 31613003

[B296] Tsurusaki Y. Okamoto N. Ohashi H. Kosho T. Imai Y. Hibi-Ko Y. (2012). Mutations affecting components of the SWI/SNF complex cause Coffin-Siris syndrome. *Nat. Genet.* 44 376–378. 10.1038/ng.2219 22426308

[B297] Tsurusaki Y. Okamoto N. Ohashi H. Mizuno S. Matsumoto N. Makita Y. (2014). Coffin-Siris syndrome is a SWI/SNF complex disorder. *Clin. Genet.* 85 548–554. 10.1111/cge.12225 23815551

[B298] Tukun A. Renda Y. Topcu M. Tuncali T. Bokesoy I. (2000). Mental retardation with rare fragile site expressed at 2q11. *Brain Dev.* 22 498–500. 10.1016/s0387-7604(00)00189-3 11111064

[B299] Uehara T. Abe K. Oginuma M. Ishitani S. Yoshihashi H. Okamoto N. (2020). Pathogenesis of CDK8-associated disorder: two patients with novel CDK8 variants and *in vitro* and *in vivo* functional analyses of the variants. *Sci. Rep.* 10:17575. 10.1038/s41598-020-74642-4 33067521 PMC7567849

[B300] van den Berg D. L. Azzarelli R. Oishi K. Martynoga B. Urban N. Dekkers D. H. (2017). Nipbl interacts with Zfp609 and the integrator complex to regulate cortical neuron migration. *Neuron* 93 348–361. 10.1016/j.neuron.2016.11.047 28041881 PMC5263256

[B301] van der, Aa N. Vandeweyer G. Kooy R. F. (2010). A boy with mental retardation, obesity and hypertrichosis caused by a microdeletion of 19p13.12. *Eur. J. Med. Genet.* 53 291–293. 10.1016/j.ejmg.2010.05.006 20570643

[B302] van Haelst M. M. Monroe G. R. Duran K. Van Binsbergen E. Breur J. M. Giltay J. C. (2015). Further confirmation of the MED13L haploinsufficiency syndrome. *Eur. J. Hum. Genet.* 23 135–138. 10.1038/ejhg.2014.69 24781760 PMC4266749

[B303] Vergano S. A. Van Der Sluijs P. J. Santen G. (1993). “ARID1B-Related disorder,” in *GeneReviews(®)*, eds Adam M. P. Ardinger H. H. Pagon R. A. Wallace S. E. Bean L. J. H. Mirzaa G. (Seattle, WA: University of Washington, Seattle). 31132234

[B304] Vervoort S. J. Welsh S. A. Devlin J. R. Barbieri E. Knight D. A. Offley S. (2021). The PP2A-Integrator-CDK9 axis fine-tunes transcription and can be targeted therapeutically in cancer. *Cell* 184 3143–3162.e32. 10.1016/j.cell.2021.04.022 34004147 PMC8567840

[B305] Vissers L. E. Van Ravenswaaij C. M. Admiraal R. Hurst J. A. De Vries B. B. Janssen I. M. (2004). Mutations in a new member of the chromodomain gene family cause CHARGE syndrome. *Nat. Genet.* 36 955–957. 10.1038/ng1407 15300250

[B306] Vogel T. Gruss P. (2009). Expression of Leukaemia associated transcription factor Af9/Mllt3 in the cerebral cortex of the mouse. *Gene Exp. Patterns* 9 83–93. 10.1016/j.gep.2008.10.004 19000783

[B307] Voisin N. Schnur R. E. Douzgou S. Hiatt S. M. Rustad C. F. Brown N. J. (2021). Variants in the degron of AFF3 are associated with intellectual disability, mesomelic dysplasia, horseshoe kidney, and epileptic encephalopathy. *Am. J. Hum. Genet.* 108 857–873. 10.1016/j.ajhg.2021.04.001 33961779 PMC8206167

[B308] von Bergh A. R. Beverloo H. B. Rombout P. Van Wering E. R. Van Weel M. H. Beverstock G. C. (2002). LAF4, an AF4-related gene, is fused to MLL in infant acute lymphoblastic leukemia. *Genes Chromosomes Cancer* 35 92–96. 10.1002/gcc.10091 12203795

[B309] Vos S. M. Farnung L. Boehning M. Wigge C. Linden A. Urlaub H. (2018a). Structure of activated transcription complex Pol II-DSIF-PAF-SPT6. *Nature* 560 607–612. 10.1038/s41586-018-0440-4 30135578

[B310] Vos S. M. Farnung L. Urlaub H. Cramer P. (2018b). Structure of paused transcription complex Pol II-DSIF-NELF. *Nature* 560 601–606. 10.1038/s41586-018-0442-2 30135580 PMC6245578

[B311] Vrouwe M. G. Elghalbzouri-Maghrani E. Meijers M. Schouten P. Godthelp B. C. Bhuiyan Z. A. (2007). Increased DNA damage sensitivity of Cornelia de Lange syndrome cells: evidence for impaired recombinational repair. *Hum. Mol. Genet.* 16 1478–1487. 10.1093/hmg/ddm098 17468178

[B312] Vulto-van Silfhout A. T. De Vries B. B. Van Bon B. W. Hoischen A. Ruiterkamp-Versteeg M. Gilissen C. (2013). Mutations in MED12 cause X-linked Ohdo syndrome. *Am. J. Hum. Genet.* 92 401–406. 10.1016/j.ajhg.2013.01.007 23395478 PMC3591845

[B313] Wade P. A. Werel W. Fentzke R. C. Thompson N. E. Leykam J. F. Burgess R. R. (1996). A novel collection of accessory factors associated with yeast RNA polymerase II. *Protein Exp. Purif.* 8 85–90. 10.1006/prep.1996.0077 8812838

[B314] Wan Y. Anastasakis D. G. Rodriguez J. Palangat M. Gudla P. Zaki G. (2021). Dynamic imaging of nascent RNA reveals general principles of transcription dynamics and stochastic splice site selection. *Cell* 184 2878–2895.e20. 10.1016/j.cell.2021.04.012 33979654 PMC8183334

[B315] Wang T. Hoekzema K. Vecchio D. Wu H. Sulovari A. Coe B. P. (2020). Large-scale targeted sequencing identifies risk genes for neurodevelopmental disorders. *Nat. Commun.* 11:4932. 10.1038/s41467-020-18723-y PMC7530681 33004838

[B316] Wang W. Yao X. Huang Y. Hu X. Liu R. Hou D. (2013). Mediator MED23 regulates basal transcription *in vivo via* an interaction with P-TEFb. *Transcription* 4 39–51. 10.4161/trns.22874 23340209 PMC3644042

[B317] Wang Y. Shen Y. Dai Q. Yang Q. Zhang Y. Wang X. (2017). A permissive chromatin state regulated by ZFP281-AFF3 in controlling the imprinted Meg3 polycistron. *Nucleic Acids Res.* 45 1177–1185. 10.1093/nar/gkw1051 28180295 PMC5388394

[B318] Weiss F. D. Calderon L. Wang Y. F. Georgieva R. Guo Y. Cvetesic N. (2021). Neuronal genes deregulated in Cornelia de Lange Syndrome respond to removal and re-expression of cohesin. *Nat. Commun.* 12:2919. 10.1038/s41467-021-23141-9 34006846 PMC8131595

[B319] Wieczorek D. Bögershausen N. Beleggia F. Steiner-Haldenstätt S. Pohl E. Li Y. (2013). A comprehensive molecular study on Coffin-Siris and Nicolaides-Baraitser syndromes identifies a broad molecular and clinical spectrum converging on altered chromatin remodeling. *Hum. Mol. Genet.* 22 5121–5135. 10.1093/hmg/ddt366 23906836

[B320] Wier A. D. Mayekar M. K. Heroux A. Arndt K. M. Vandemark A. P. (2013). Structural basis for Spt5-mediated recruitment of the Paf1 complex to chromatin. *Proc. Natl. Acad. Sci. U S A.* 110 17290–17295. 10.1073/pnas.1314754110 24101474 PMC3808610

[B321] Willemsen R. Bontekoe C. J. Severijnen L. A. Oostra B. A. (2002). Timing of the absence of FMR1 expression in full mutation chorionic villi. *Hum. Genet.* 110 601–605. 10.1007/s00439-002-0723-5 12107447

[B322] Winter G. E. Mayer A. Buckley D. L. Erb M. A. Roderick J. E. Vittori S. (2017). BET bromodomain proteins function as master transcription elongation factors independent of CDK9 recruitment. *Mol. Cell.* 67 5–18.e19. 10.1016/j.molcel.2017.06.004 28673542 PMC5663500

[B323] Wu S. Y. Chiang C. M. (2007). The double bromodomain-containing chromatin adaptor Brd4 and transcriptional regulation. *J. Biol. Chem.* 282 13141–13145. 10.1074/jbc.R700001200 17329240

[B324] Wu S. Y. Lee A. Y. Lai H. T. Zhang H. Chiang C. M. (2013). Phospho switch triggers Brd4 chromatin binding and activator recruitment for gene-specific targeting. *Mol. Cell.* 49 843–857. 10.1016/j.molcel.2012.12.006 23317504 PMC3595396

[B325] Wu S. Fatkhutdinov N. Rosin L. Luppino J. M. Iwasaki O. Tanizawa H. (2019). ARID1A spatially partitions interphase chromosomes. *Sci. Adv.* 5:eaaw5294. 10.1126/sciadv.aaw5294 31131328 PMC6531001

[B326] Xiang Y. Tanaka Y. Patterson B. Hwang S. M. Hysolli E. Cakir B. (2020). Dysregulation of BRD4 function underlies the functional abnormalities of MeCP2 mutant neurons. *Mol. Cell.* 79 84–98.e9. 10.1016/j.molcel.2020.05.016 32526163 PMC7375197

[B327] Xu Y. Vakoc C. R. (2017). Targeting cancer cells with BET bromodomain inhibitors. *Cold Spring Harb. Perspect. Med.* 7:a026674. 10.1101/cshperspect.a026674 28213432 PMC5495050

[B328] Xu Y. Milazzo J. P. Somerville T. D. D. Tarumoto Y. Huang Y. H. Ostrander E. L. (2018). A TFIID-SAGA perturbation that targets MYB and suppresses acute myeloid leukemia. *Cancer Cell* 33 13–28.e8. 10.1016/j.ccell.2017.12.002 29316427 PMC5764110

[B329] Yadav D. Ghosh K. Basu S. Roeder R. G. Biswas D. (2019). Multivalent role of human TFIID in recruiting elongation components at the promoter-proximal region for transcriptional control. *Cell Rep.* 26 1303–1317.e7. 10.1016/j.celrep.2019.01.012 30699356 PMC6368250

[B330] Yin J. W. Wang G. (2014). The mediator complex: a master coordinator of transcription and cell lineage development. *Development* 141 977–987. 10.1242/dev.098392 24550107

[B331] Yokoyama A. Lin M. Naresh A. Kitabayashi I. Cleary M. L. (2010). A higher-order complex containing AF4 and ENL family proteins with P-TEFb facilitates oncogenic and physiologic MLL-dependent transcription. *Cancer Cell* 17 198–212. 10.1016/j.ccr.2009.12.040 20153263 PMC2824033

[B332] Yu M. Yang W. Ni T. Tang Z. Nakadai T. Zhu J. (2015). RNA polymerase II-associated factor 1 regulates the release and phosphorylation of paused RNA polymerase II. *Science* 350 1383–1386. 10.1126/science.aad2338 26659056 PMC8729149

[B333] Yuan B. Pehlivan D. Karaca E. Patel N. Charng W. L. Gambin T. (2015). Global transcriptional disturbances underlie Cornelia de Lange syndrome and related phenotypes. *J. Clin. Invest.* 125 636–651. 10.1172/JCI77435 25574841 PMC4319410

[B334] Zarate Y. A. Uehara T. Abe K. Oginuma M. Harako S. Ishitani S. (2021). CDK19-related disorder results from both loss-of-function and gain-of-function de novo missense variants. *Genet. Med.* 23 1050–1057. 10.1038/s41436-020-01091-9 33495529

[B335] Zeitlinger J. Stark A. Kellis M. Hong J. W. Nechaev S. Adelman K. (2007). RNA polymerase stalling at developmental control genes in the *Drosophila melanogaster* embryo. *Nat. Genet.* 39 1512–1516. 10.1038/ng.2007.26 17994019 PMC2824921

[B336] Zhang C. Mejia L. A. Huang J. Valnegri P. Bennett E. J. Anckar J. (2013). The X-linked intellectual disability protein PHF6 associates with the PAF1 complex and regulates neuronal migration in the mammalian brain. *Neuron* 78 986–993. 10.1016/j.neuron.2013.04.021 23791194 PMC3694281

[B337] Zhang W. Xia X. Reisenauer M. R. Hemenway C. S. Kone B. C. (2006). Dot1a-AF9 complex mediates histone H3 Lys-79 hypermethylation and repression of ENaCalpha in an aldosterone-sensitive manner. *J. Biol. Chem.* 281 18059–18068. 10.1074/jbc.M601903200 16636056 PMC3015183

[B338] Zhang X. Wang Y. Yang F. Tang J. Xu X. Yang L. (2020). Biallelic INTS1 mutations cause a rare neurodevelopmental disorder in two chinese siblings. *J. Mol. Neurosci.* 70 1–8. 10.1007/s12031-019-01393-x 31428919

[B339] Zhang Y. Wang C. Liu X. Yang Q. Ji H. Yang M. (2019). AFF3-DNA methylation interplay in maintaining the mono-allelic expression pattern of XIST in terminally differentiated cells. *J. Mol. Cell Biol.* 11 761–769. 10.1093/jmcb/mjy074 30535390 PMC7727261

[B340] Zheng B. Aoi Y. Shah A. P. Iwanaszko M. Das S. Rendleman E. J. (2021). Acute perturbation strategies in interrogating RNA polymerase II elongation factor function in gene expression. *Genes Dev.* 35 273–285. 10.1101/gad.346106.120 33446572 PMC7849361

[B341] Zheng H. Qi Y. Hu S. Cao X. Xu C. Yin Z. (2020). Identification of Integrator-PP2A complex (INTAC), an RNA polymerase II phosphatase. *Science* 370:eabb5872. 10.1126/science.abb5872 33243860

[B342] Zhou H. Kim S. Ishii S. Boyer T. G. (2006). Mediator modulates Gli3-dependent Sonic hedgehog signaling. *Mol. Cell. Biol.* 26 8667–8682. 10.1128/MCB.00443-06 17000779 PMC1636813

[B343] Zhou H. Spaeth J. M. Kim N. H. Xu X. Friez M. J. Schwartz C. E. (2012). MED12 mutations link intellectual disability syndromes with dysregulated GLI3-dependent Sonic Hedgehog signaling. *Proc. Natl. Acad. Sci. U S A.* 109 19763–19768. 10.1073/pnas.1121120109 23091001 PMC3511715

[B344] Zuin J. Franke V. Van Ijcken W. F. Van Der Sloot A. Krantz I. D. Van Der Reijden M. I. (2014). A cohesin-independent role for NIPBL at promoters provides insights in CdLS. *PLoS Genet.* 10:e1004153. 10.1371/journal.pgen.1004153 PMC3923681 24550742

